# Activation of a FOXO3-induced cell cycle arrest regulates ferroptosis

**DOI:** 10.1038/s41420-025-02760-x

**Published:** 2025-10-16

**Authors:** Huanjie Huang, Matthias van Sligtenhorst, Alida M. M. Smits, Can Gulersonmez, Edwin Stigter, Tobias B. Dansen, Boudewijn M. T. Burgering

**Affiliations:** 1 Oncode Institute, University Medical Center Utrecht, Utrecht, The Netherlands; 2 Center for Molecular Medicine, University Medical Center Utrecht, Utrecht, The Netherlands; 3 Present Address: Department of Chronic Diseases and Metabolism, KU Leuven, Leuven, Belgium

**Keywords:** Cell death, Transcription, Stress signalling

## Abstract

FOXO transcription factors act downstream of PI3K signaling, and FOXO transcriptional activity is inhibited through nuclear exclusion by PKB/AKT-mediated phosphorylation. Many studies have shown FOXO to contribute to organismal homeostasis by mitigating (extra)cellular stress to prevent cell death (reviewed in [1]). Here we show that FOXO3 activation protects cells from ferroptosis, an iron-dependent form of non-apoptotic cell death. In untransformed hTERT-RPE-1 cells, FOXO3 activation reduces ferroptosis in a multilayered manner. First, FOXO3 mediates protection from ferroptosis in part through a p27-induced G1 cell cycle arrest. Second, FOXO3 activation reduces cellular H_2_O_2_ levels, thereby limiting substrate availability for the Fenton reaction, which fuels hydroxyl radical formation for lipid peroxidation. Third, FOXO3 activation lowers cellular iron content by reducing TFR1 expression, which, combined with the lowering of cellular H_2_O_2_ levels, likely further reduces the formation of hydroxyl radicals through the Fenton reaction. Finally, FOXO3 activation reduces expression of long-chain-fatty-acid—CoA ligase 4 (ACSL4) and Peroxisomal targeting signal 1 receptor (PEX5), proteins involved in lipid metabolism and protection against ferroptosis. Taken together, we show that FOXO3 activation results in protection from ferroptosis, adding to the repertoire of FOXO-controlled cell protection programs.

## Introduction

Cells that function normally need to be protected from cell death. Yet, mechanisms whereby cells are protected from cell death may differ. This also holds for proliferating and non-proliferating cells, where protective mechanisms preventing cell death differ. Importantly, signaling pathways that regulate the transition between proliferative and non-proliferative states also regulate survival mechanisms, thereby ensuring a hardwired connection between survival mechanism and cell state. PI3K signaling serves as a typical example in this respect. Active PI3K signaling drives cell proliferation through several downstream pathways, including PKB/AKT. PKB/AKT regulates proliferation through negative regulation of cell cycle inhibitors (p27kip1 (ref. [1])) and Forkhead box O (FOXO) transcription factors (reviewed in [2]). On the other hand, PKB/AKT also regulates cell survival, for example, by preventing apoptosis induction by inhibitory phosphorylation of BAD [3], but also by preventing proteotoxicity [4].

By now, it is apparent that there are many ways for a cell to die. Whereas initially cell death was considered to result from either apoptosis (also known as programmed cell death) or necrosis (uncontrolled cell death), recently various other forms of cell death have been described (reviewed in e.g., [5]). Ferroptosis was originally described as an iron-dependent form of non-apoptotic cell death induced by the small molecule erastin, with specificity towards KRAS mutant cancer cell lines [6]. Cell death by ferroptosis is due to iron-dependent accumulation of peroxidised polyunsaturated fatty acid phospholipids (PUFA-PL-OOH). Lipid oxidation is caused by reactive oxygen species (ROS), in particular hydroxyl radicals. Therefore, conditions that increase ROS can potentially induce ferroptosis. In this respect, the small molecule erastin was shown to inhibit the plasma membrane localized cystine-glutamate antiporter system x_c_- [7, 8]. System x_c_- is a heterodimer consisting of SLC7A11 (xCT) and 4F2hc (also known as CD98 or FRP1) that mediates the exchange of extracellular cystine for intracellular glutamate. Cystine is reduced intracellularly to cysteine, which can be metabolized to produce glutathione. By inhibiting system x_c_-, erastin lowers cellular reductive capacity by lowering cellular glutathione levels. This lowering of cellular reductive capacity may result in increased cellular ROS levels and hence sensitivity to ferroptosis induction. Moreover, GPX4, the main scavenger of lipid hydroperoxides, requires glutathione to reduce PUFA-PL-OOH to non-toxic lipid alcohols (PUFA-PL-OH). Indeed, inhibitors of GPX4 like RAS-selective lethal compound 3 (RSL3, ref. [9]) induce ferroptosis.

In line with PI3K/AKT signaling, enabling cell survival of proliferating cells by inhibiting apoptosis through various mechanisms, PKB/AKT also inhibits ferroptosis by regulating lipid synthesis through mTOR/SREBP [10] or through Creatine Kinase B [11]. Thus, cells require PI3K signaling to PKB/AKT to stimulate proliferation and to protect proliferating cells from various forms of cell death, including ferroptosis.

PKB/AKT phosphorylates and inhibits Forkhead box O (FOXO) transcription factors. The FOXO family consists of four members, FOXO1, FOXO3, FOXO4, and FOXO6, that are functionally similar, but displaying tissue-specific expression (for a recent review and discussion see [12]). FOXO transcription factors are the mammalian orthologues of the Caenorhabditis elegans transcription factor DAF-16. DAF-16 was identified as the first gene that, when activated, prolongs lifespan by controlling a reversible developmental arrest (diapause) that enables survival under stress conditions [13]. Taken together, this indicates that an evolutionary conserved role of DAF-16/FOXOs is to regulate survival by inhibiting proliferation and providing at the same time coping strategies to prevent (cell) death.

Similar to the DAF-16-dependent developmental arrest of *C. elegans*, FOXOs induce cell cycle arrest in mammalian cells, and PKB/AKT regulates cell cycle progression by phosphorylation of FOXO [14]. Importantly, whereas PKB/AKT drives cell proliferation and mediates protection from cell death, FOXOs halt proliferation and mediate protection of thereby arrested cells from oxidative stress-induced apoptosis [15].

Here, we studied the role of FOXOs, more specifically FOXO3 in ferroptosis and we used the hTERT-immortalized retinal pigment epithelial cell line HTERT-RPE-1 as this cell line. Activation of FOXO3 in HTERT-RPE-1 cells provides resistance to ferroptosis that depends on a cell cycle arrest in G1, as well as on FOXO-dependent regulation of iron metabolism and increased scavenging of H_2_O_2_. Combined, these likely limits formation of hydroxyl radicals responsible for lipid oxidation. In agreement, FOXO3 activation lowers C11-BODIPY^581/591^ oxidation, a surrogate read-out for lipid peroxidation. Finally, we show that FOXO3 activation impacts lipid metabolism linked to ferroptosis through reducing the expression of ACSL4 and PEX5 (refs. [16, 17]).

Taken together, our results show that active PI3K signaling regulates sensitivity to ferroptosis in proliferative conditions through PKB/AKT and in non-proliferative conditions through FOXO activation in the absence of PI3K signaling.

## Results

### FOXO3 activation protects against ferroptosis

**Fig. 1:**
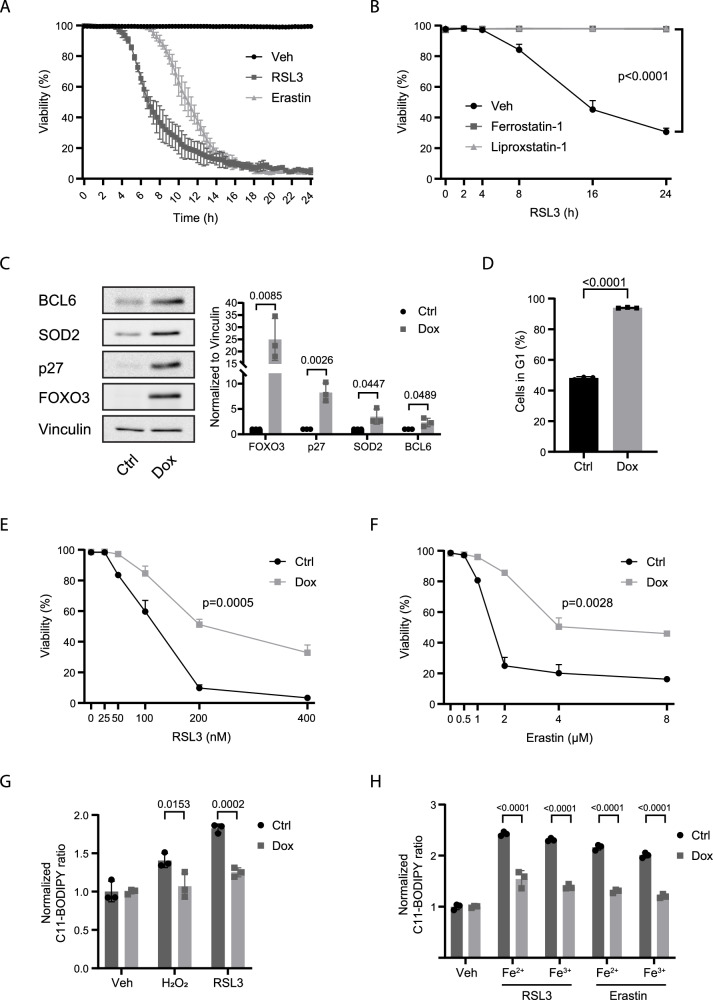
FOXO3 activation protects hTERT-RPE-1 cells against ferroptosis. **A** hTERT-RPE-1 cells were treated with a vehicle, 200 nM RSL3, or 5 µM erastin prior to live imaging. (n = 3). **B** Flow cytometry analysis of hTERT-RPE-1 cell viability after treatment with 50 nM RSL3 in a time-dependent manner, 500 nM ferrostatin-1, or 200 nM liproxstatin-1 was simultaneously added to the medium along with RSL3. (n = 3, unpaired t test with significant differences from time point 24 h). **C** Characterization of hTERT-RPE-1 F3A3 cells after FOXO3 activation. Protein expression was analyzed by Western blot. (n = 3, unpaired t test). **D** Flow cytometry analysis of cell cycle using FUCCI cell cycle reporter. hTERT-RPE-1 F3A3 cells were either left untreated or treated with doxycycline for 24 h. (n = 3, unpaired t test). **E**, **F** FOXO3.A3 expression protects against ferroptosis. hTERT-RPE-1 F3A3 cells were either left untreated or treated with doxycycline for 24 h, and thereafter cells were treated for 8 h with indicated dose of RSL3 (**E**, n = 3, unpaired t test with significant differences from dose 200 nM) or for 16 h with indicated doses of erastin (**F**, n = 3, unpaired t test with significant differences from dose 4 µM). **G**, **H** FOXO3 activation reduces lipid peroxidation. hTERT-RPE-1 F3A3 cells were either left untreated or treated with doxycycline for 24 h, and thereafter cells were loaded with C11-BODIPY^581/591^ probe and subsequently left untreated or treated for 5 h with 100 µM H_2_O_2_, 200 nM RSL3, or 4 µM erastin. Concomitant treatment with 100 µM Fe^2+^ or Fe^3+^ was used to enhance lipid peroxidation. C11-BODIPY^581/591^ oxidation was determined as described under materials and methods. (n = 3, one-way ANOVA with significant differences between Ctrl and Dox groups).

Control of the cellular stress response is an evolutionary conserved function of FOXO activation in normal physiology [12]. Therefore, we chose to study a possible role for FOXO in the control of ferroptosis in the hTERT-immortalized human retinal pigment epithelial (hTERT-RPE-1) cell line. hTERT-RPE-1 cells have a stable karyotype with an average chromosome number of 46 and have been widely used to study cellular processes in a non-cancer setting. The process of epithelial to mesenchymal transition (EMT) has been suggested to render cells sensitive to ferroptosis, suggesting that epithelial cells could be insensitive to ferroptosis [18, 19]. Therefore, we first tested whether hTERT-RPE-1 cells are sensitive to ferroptosis. Treatment of hTERT-RPE-1 cells with the GPX4 inhibitor RSL3 (ref. [20]) or the antiporter system x_c-_ inhibitor erastin [21] resulted in a time-dependent induction of cell death (Fig. 1A, B). This could be rescued by pretreatment of cells with ferrostatin-1, a scavenger of alkoxyl radicals, or liproxstatin-1, which suppresses ferroptosis by subverting the lipid peroxidation process. This indicates that hTERT-RPE-1 cells are sensitive to ferroptosis. To further characterize the ferroptosis response in RPE-1 cells, we analyzed RNA and protein expression of known critical regulators of ferroptosis (ACSL4, GPX4, SLC7A11, ferritin, PEX5, TFR1) following RSL3 or erastin treatment. (Supplementary Fig. 1A, B). RSL3 or erastin treatment did not induce any significant change in RNA or protein expression of these ferroptosis regulators, except for SLC7A11. The latter one is regulated at the RNA but not at the protein level by erastin. We also analyzed whether RSL3 increased Malondialdehyde (MDA), an endpoint of lipid peroxidation. Indeed, RSL3 increased MDA levels (Supplementary Fig. 1C).

To test whether FOXO activation affects ferroptotic cell death induced by RSL3 or erastin, we generated RPE cells that stably express a FOXO3 allele mutated at the PKB/AKT phosphorylation sites (FOXO3.A3) under the control of a doxycycline-inducible promoter, hereafter referred to as hTERT-RPE-1 F3A3 cells. As reported previously for other cell types, FOXO3 activation induced expression of p27kip1 (ref. [14]), MnSOD/SOD2 (ref. [15]), and BCL6 (ref. [22]) (Fig. 1C, and Supplementary Fig. 1D), which is accompanied by a cell cycle arrest in G1 (Fig. 1D and [14]). Importantly, following induction of FOXO3.A3 expression, RSL3-induced cell death was significantly reduced, suggesting that FOXO3 activation protects against ferroptosis (Fig. 1E). A similar reduction in ferroptosis upon FOXO3.A3 expression was observed in response to erastin treatment (Fig. 1F). Cellular stress, in particular oxidative stress, has been shown to induce FOXO translocation to the nucleus [23–25]; therefore, to study whether ferroptosis induction regulates FOXO, we tested whether RSL3 or erastin treatment induced FOXO nuclear translocation. However, during the time course tested, we did not observe translocation of either FOXO3 or FOXO1 (Supplementary Fig. 1E, F). We also assessed phosphorylation of AKT/PKB (ser473), which is indicative for PKB/AKT activation, and phosphorylation of FOXO3 (thr32) and FOXO1 (thr24), which indicates FOXO inhibition due to cytosolic relocation. No significant changes were observed, arguing that RSL3 or erastin also did not affect PKB/AKT signaling towards FOXOs (Supplementary Fig. 1B). Apparently, induction of ferroptosis is not accompanied by a (failed) rescue response, as we do not observe increased expression of known anti-ferroptosis regulators (Supplementary Fig. 1A, B) or activation of FOXOs (Supplementary Fig. 1B, E, F). Ferroptosis proceeds through lipid peroxidation, and this can be measured using oxidation of the C11-BODIPY^581/591^ probe as a proxy. Treatment of hTERT-RPE-1 cells with H_2_O_2_, RSL3, and erastin induced C11-BODIPY^581/591^ oxidation, which was further enhanced by concomitant Fe^2+^/^3+^ treatment, indicating that these treatments indeed induce lipid peroxidation characteristic for ferroptosis. Importantly, FOXO3 activation consistently lowered C11-BODIPY^581/591^ oxidation under all conditions (Fig. 1G, H). To corroborate these findings, we also measured levels of MDA after FOXO3.A3 induction, and we observed the increase of MDA after RSL3 treatment to be reduced to baseline after FOXO3.A3 activation (Supplementary Fig. 1C).

Thus, combined these data indicate that FOXO3 activation reduces the sensitivity of hTERT-RPE-1 cells to ferroptosis.

### FOXO3-induced G1 arrest mediates control of ferroptosis

**Fig. 2:**
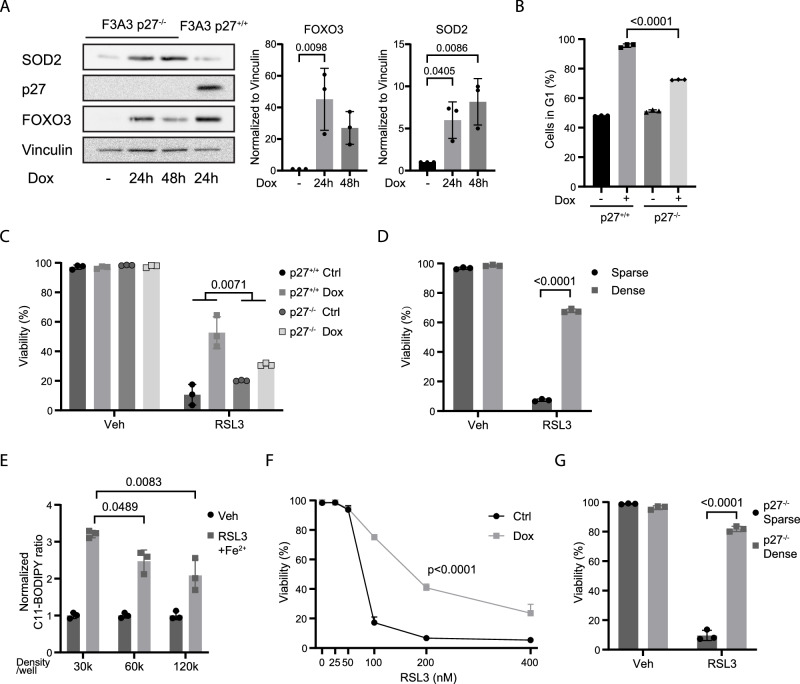
FOXO3 activation does not protect against ferroptosis through contact inhibition. **A** Characterization of hTERT-RPE-1 F3A3 p27-/- cell line after FOXO3 activation. Cells were treated with doxycycline for 24 or 48 h, and expression of FOXO targets was analyzed by Western blot. Total cell lysate of wild-type hTERT-RPE-1 F3A3 cells was used to determine band height of p27. (n = 3, one-way ANOVA). **B** Cell cycle profile of hTERT-RPE-1 F3A3 WT and hTERT-RPE-1 F3A3 p27-/- cells after either untreated or treated with doxycycline for 24 h and analyzed by FACS. (n = 3, one-way ANOVA). **C** hTERT-RPE-1 F3A3 WT and hTERT-RPE-1 F3A3 p27-/- cells were either left untreated or treated with doxycycline for 24 h, followed by an 8 h treatment with either a vehicle or 200 nM RSL3. The difference in cell viability between doxycycline-treated and untreated conditions was defined as the rescue percentage (Viability_dox_−Viability_Ctrl_). (n = 3, unpaired t test with significant differences in rescue percentage between WT and p27-/- cells). **D** hTERT-RPE-1 cells were plated at spare or dense density, followed by an 8 h treatment with either vehicle or 200 nM RSL3 and analyzed by FACS. (n = 3, one-way ANOVA). **E** HTERT-RPE-1 cells were plated at indicated seeding density. 200 nM RSL3 and 100 µM Fe^2+^ were used to induce lipid peroxidation. (n = 3, one-way ANOVA). **F** hTERT-RPE-1 F3A3 cells were plated at sparse density. Cells were either left untreated or treated with doxycycline for 24 h and thereafter treated for 8 h with indicated dose of RSL3. (n = 3, unpaired t test with significant differences from dose 200 nM). **G** hTERT-RPE-1 p27-/- cells were plated at sparse or dense confluency, followed by an 8 h treatment with either vehicle or 200 nM RSL3 and analyzed by flow cytometry. (n = 3, one-way ANOVA).

FOXO3 activation induces a G1 cell cycle arrest, and it has recently been reported that a G1 cell cycle arrest in various cancer cell lines renders these cells resistant to ferroptosis [26]. The FOXO3.A3-induced G1 cell cycle arrest is dependent on the cell cycle inhibitor p27kip1(ref. [14]). We therefore tested whether FOXO3 protects cells in p27kip1 knock-out hTERT-RPE-1 cells from ferroptosis (referred to as hTERT-RPE-1 F3A3 p27-/-). Both the FOXO3.A3-induced G1 arrest as well as ferroptosis resistance were impaired in hTERT-RPE-1 F3A3 27-/- (Fig. 2A–C). This suggests that the p27kip1-mediated G1 arrest contributes to FOXO3-induced ferroptosis protection.

High cell density also causes a G1 arrest, through contact inhibition, and has been shown to protect against ferroptosis [27]. Indeed, also for hTERT-RPE-1 cells cell density induced contact inhibition resulted in robust protection from RSL3-induced ferroptosis (Fig. 2D) and reduced C11-BODIPY^581/591^ oxidation (Fig. 2E). To determine whether FOXO3-induced G1 arrest and ferroptosis protection relates to cell density and contact inhibition we analyzed FOXO3-mediated protection at low cell density and observed this to be independent of cell density (Fig. 2F). Furthermore, whereas deletion of p27kip1 impaired FOXO3-induced ferroptosis protection, hTERT-RPE-1 F3A3 p27-/- cells grown at high density did show robust ferroptosis protection (Fig. 2G). Thus, it appears that a G1 arrest rather than p27 contributes to FOXO3-mediated ferroptosis protection.

### FOXO3-mediated control of lipid metabolism contributes to ferroptosis protection

**Fig. 3:**
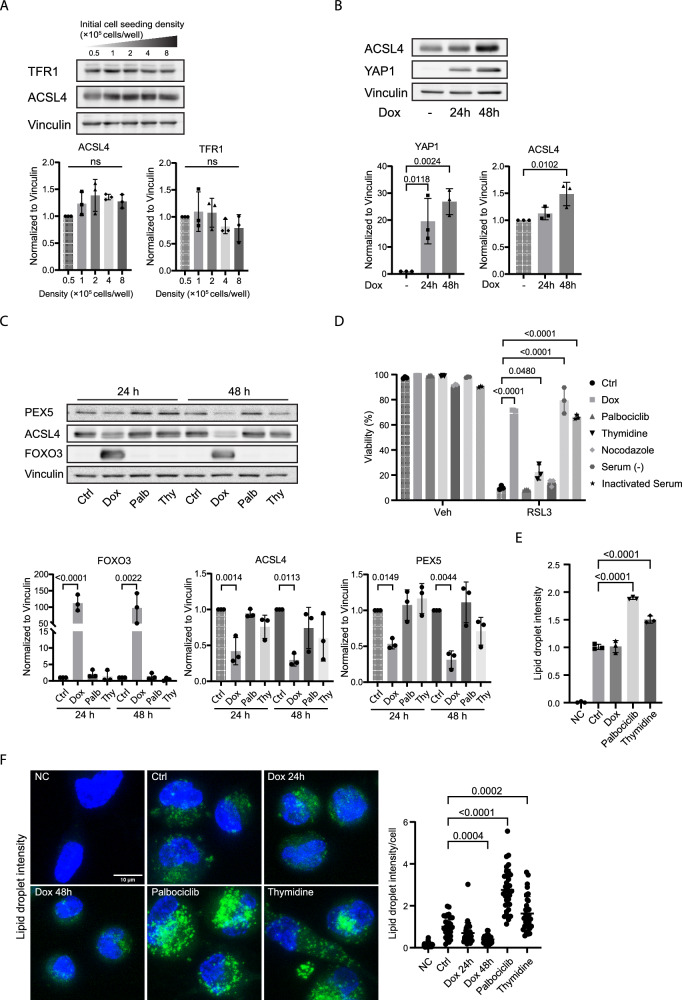
FOXO3-mediated regulation of lipid metabolism. **A** hTERT-RPE-1 cells were plated at indicated seeding density for 48 h, protein expression was analyzed by Western blot. (ns: non-significant). (n = 3, one-way ANOVA). **B** hTERT-RPE-1 YAP1 S5A cells were treated with doxycycline for 24 or 48 h, protein expression was analyzed by Western blot. (n = 3, one-way ANOVA). **C** FOXO3 activation and thymidine treatment induced arrest reduce expression of proteins involved in lipid metabolism. hTERT-RPE-1 F3A3 cells were treated with indicated compounds for 24 or 48 h, protein expression was analyzed by Western blot. (Palb palbociclib, Thy thymidine). (n = 3, one-way ANOVA). **D** Induction of hTERT-RPE-1 cell cycle arrest by various treatments results in variable ferroptosis protection. Cell cycle arrest was induced with indicated treatments for 24 h. Ferroptosis sensitivity was determined by treating cells with 200 nM RSL3 for 8 h and analyzed by flow cytometry. (Serum (-): serum deprivation; Inactivated Serum: 95 °C heat-inactivated serum). (n = 3, one-way ANOVA). **E** hTERT-RPE-1 F3A3 cells were pretreated with indicated compounds for 24 h, then loaded with BODIPY 493/503 before flow cytometry. (n = 3, one-way ANOVA). **F** hTERT-RPE-1 F3A3 cells were pretreated with indicated compounds for 24 or 48 h, then loaded with BODIPY 493/503 before microscopy (left panel), quantification, and statistical analysis were shown (right panel). (n = 3, one-way ANOVA).

Cellular contact inhibition through E-cadherin/YAP1 signaling regulates expression of acyl-CoA synthetase long chain family member 4 (ACSL4) and transferrin receptor 1 (TFR1), which are critical mediators of ferroptosis sensitivity [16, 28]. However, we did not observe regulation of ACSL4 or TFR1 expression following density arrest (Fig. 3A). hTERT-RPE-1 cells are not refractory to YAP signaling because inducible expression of a constitutive active YAP1 mutant, YAPS5A [29], did increase ACSL4 expression indicating that in hTERT-RPE-1 cells ACSL4 expression can be controlled by YAP1 (Fig. 3B). In contrast to increased cell density, FOXO3.A3 expression downregulated ACSL4 expression (Fig. 3C). Next to ACSL4, peroxisomes are important in lipid oxidation and expression of the peroxisomal targeting receptor PEX5 has also been shown to mediate ferroptosis sensitivity [17]. In agreement, we observed next to a reduction in ACSL4 expression, also a reduction of PEX5 expression, following FOXO3 activation.

Recent evidence indicates that next to regulation of ACSL4 expression redirection of lipid metabolism during G1 arrest can mediate resistance to ferroptosis through lipid droplet formation [26].

Therefore, we also tested other treatments known to induce a cell cycle arrest in G1 to test how these compare to FOXO3-induced arrest and ferroptosis inhibition (Fig. 3D). We observed that both serum deprivation and using heat-inactivated serum (95 °C) provided strong protection against ferroptosis. Previously, it has been shown that in these cell culture conditions a lack of transferrin mediates ferroptosis protection [28]. This suggests that for hTERT-RPE-1 cells iron is a major determinant in hydroxyl radical formation in hTER-RPE-1 cells. A thymidine block (arrest in late G1/early S phase) resulted in minimal but significant reduction of RSL3-induced cell death. In contrast, palbociclib (a CDK4/CDK6 inhibitor) treatment induced a G1 arrest without affecting RSL3-induced ferroptosis. Palbociclib-induced G1 arrest was previously shown to mediate ferroptosis protection through lipid droplet formation [26]. To study whether lipid droplet formation contributes to ferroptosis resistance in hTERT-RPE-1 cells, we stained cells with BODIPY 493/503 (Fig. 3E, F). Surprisingly, we observed indeed a strong induction of lipid droplet formation after palbociclib treatment, but overall, no correlation between increased lipid droplet formation and resistance to ferroptosis. More importantly, FOXO3 activation did not significantly affect lipid droplet formation in hTERT-RPE-1 cells. These data suggest that in hTERT-RPE-1 cells, regulation of lipid droplet formation per se may not act as a mechanism to mediate ferroptosis resistance.

### FOXO3-mediated control of ferroptosis correlates to redox control

**Fig. 4:**
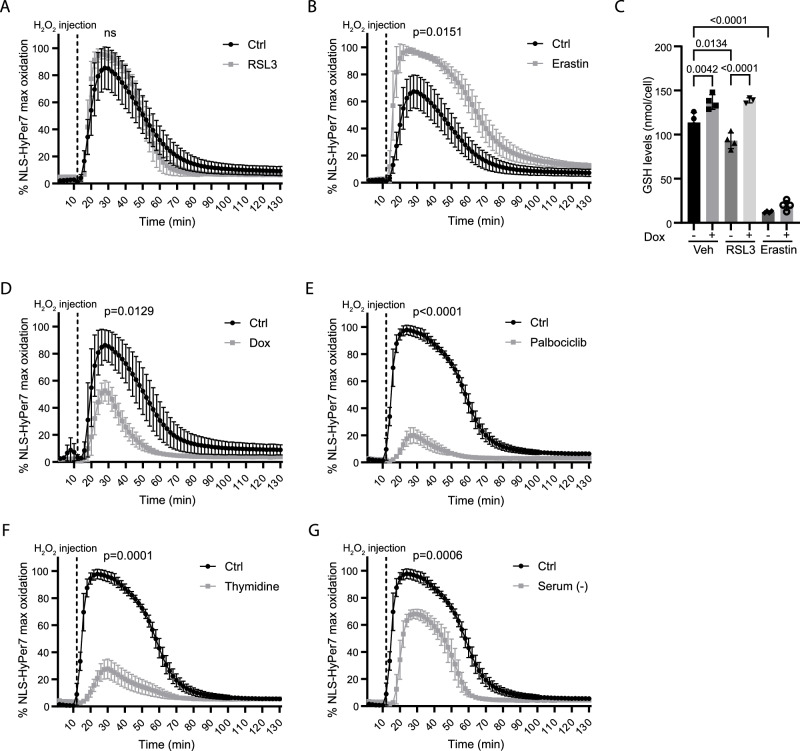
G1 cell cycle arrest increased cellular reductive capacity. **A**, **B** H_2_O_2_ measurements using the ratio metric HyPer7 sensor to determine cellular reductive capacity. hTERT-RPE-1 NLS-HyPer7 F3A3 cells were treated with indicated compounds for 24 h (left panel, 100 nM RSL3, right panel 1 µM erastin, 200 nM Liproxstatin-1 was used to inhibit cell death), followed by the addition of 50 μM H_2_O_2_. Hyper7 oxidation was measured in time. Data are from one representative experiment, and the traces are deconvoluted for clarity. (n = 3, unpaired t test with significant differences from time point 14). **C** LC-MS analysis of cellular glutathione levels. hTERT-RPE-1 NLS-HyPer7 F3A3 cells were either left untreated or treated with doxycycline for 24 h, followed by the treatment of indicated compounds (50 nM RSL3 for 4 h, 1 µM erastin for 6 h). Then, samples were collected for LC-MS analysis. (n = 3, one-way ANOVA). **D** hTERT-RPE-1 NLS-HyPer7 F3A3 cells were left untreated (Ctrl) or treated with doxycycline (Dox) for 24 h. Cells were then treated with 50 µM H_2_O_2_, and Hyper7 oxidation was measured in time. (n = 3, unpaired t test with significant differences from time point 14). **E**–**G** Same as in (**C**), but now cells were arrested by treatment with Palbociclib or Thymidine, or arrested by serum deprivation for 24 h. Cells were then treated with 50 µM H_2_O_2_, and Hyper7 oxidation was measured in time. Data are from one representative experiment, and the traces are deconvoluted for clarity. (Serum (-): serum deprivation). (n = 3, unpaired t test with significant differences from time point 14).

Ferroptosis is driven by lipid peroxidation mostly initiated by hydroxyl radicals. Hydrogen peroxide serves as major source of hydroxyl radical generation. The conversion of H_2_O_2_ to hydroxyl radicals is catalyzed by ferrous iron (Fe^2+^) through the so-called Fenton reaction [30]. Recently, sensitive genetically encoded sensors for H_2_O_2_ have been developed (HyPer7 (ref. [31])). We previously generated hTERT-RPE-1 cells expressing the HyPer7 sensor either localized in the nucleus (HyPer7-NLS) or in the cytosol (HyPer7-NES) [32]. RSL3 and erastin treatment did not directly affect cellular H_2_O_2_ levels as indicated by lack of Hyper7 oxidation; however, erastin, but not RSL3 pretreatment, did increase Hyper7 oxidation following a subsequent H_2_O_2_ challenge (Fig. 4A, B, and Supplementary Fig. 2A, B). Erastin inhibits cystine import and consequently lowers intracellular cysteine, leading to an apparent strong reduction of reduced glutathione levels (Fig. 4C). Therefore, these results suggest that a strong reduction in the cellular glutathione level, next to impairing GPX4 activity, also contributes to ferroptosis by sensitizing cells to endogenous perturbations of redox (H_2_O_2_) metabolism. FOXO3.A3 expression increased cellular glutathione levels (Fig. 4C), and in agreement, we observed that FOXO3.A3 expression significantly reduced both cytosolic and nuclear HyPer7 oxidation following treatment of cells with H_2_O_2_ (Fig. 4D and Supplementary Fig. 2C). This combined suggests that lowering of cellular H_2_O_2_ levels contributes to FOXO3-mediated ferroptosis protection.

We also tested the effect of the other treatments, resulting in a cell cycle arrest on Hyper7 oxidation. Interestingly, we observed that all treatments, including palbociclib treatment, resulted in reduced HyPer7 oxidation, after a subsequent challenge of cells with H_2_O_2_, albeit to varying extent (Fig. 4E–G and Supplementary Fig. 2D–F). This indicates that cell cycle arrest, per se, results in increased cellular reductive capacity and consequent lowering of H_2_O_2_ levels. Thus, a cell cycle arrest, likely accompanied by reduced requirement for biosynthesis, provides cells with an increased spare reductive capacity. Importantly, as we do not observe clear protection from ferroptosis by, e.g., palbociclib treatment of hTERT-RPE-1 cells, this suggests that the ability of FOXO3, or treatments that induce cell cycle arrest, to lower cellular H_2_O_2_ may contribute to ferroptosis protection, but that this is not sufficient.

### FOXO3 control of Iron metabolism

As the Fenton reaction, generating hydroxyl radicals from H_2_O_2_, is catalyzed by Fe^2+^, we reasoned that increased reductive capacity should be accompanied by proper regulation of iron metabolism to maximally reduce generation of hydroxyl radicals. Therefore, we tested the effect of FOXO3.A3 expression on iron metabolism.

**Fig. 5:**
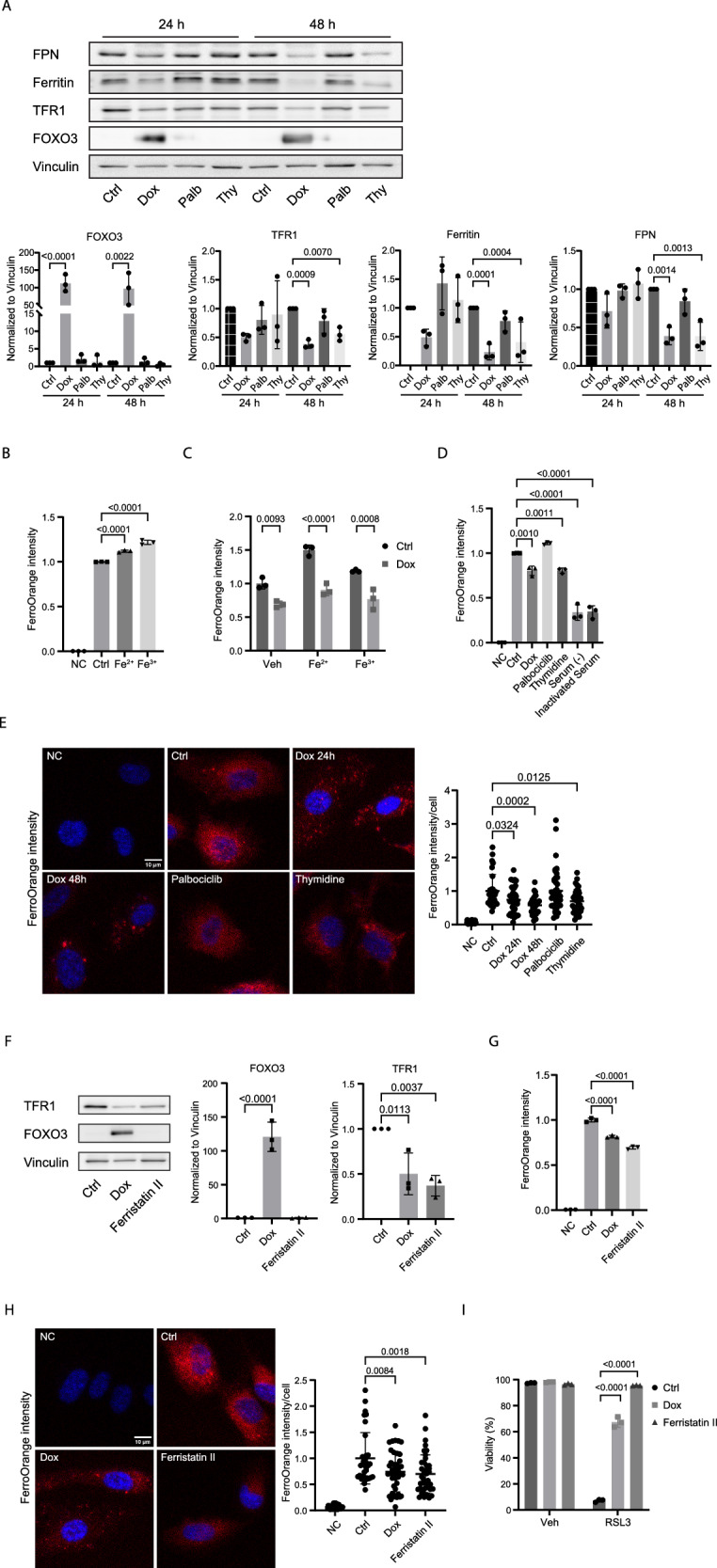
FOXO3 activation reduces cellular iron. **A** FOXO3 activation and thymidine treatment induced arrest regulate iron homeostasis. hTERT-RPE-1 F3A3 cells were treated with indicated compounds for 24 or 48 h, protein expression was analyzed by Western blot. (Palb: palbociclib; Thy: thymidine). (n = 3, one-way ANOVA). **B**, **C** Intracellular Fe^2+^ was detected by FerroOrange live cell dye. hTERT-RPE-1 F3A3 cells were either left untreated or treated with doxycycline for 24 h. The incubation of 100 µM Fe^2+^/Fe^3+^ for 30 min prior to FerroOrange staining was used as a positive control. Cells were then collected and measured by flow cytometry. (n = 3; one-way ANOVA). **D** Same as in (**C**), cells were also arrested by other treatments for 24 h, as indicated. After FerroOrange incubation, cells were then collected and measured by flow cytometry. (Serum (-): serum deprivation; Inactivated Serum: 95 °C heat-inactivated serum). (n = 3, one-way ANOVA). **E** hTERT-RPE-1 F3A3 cells were pretreated with indicated compounds for 24 or 48 h, followed by FerroOrange staining for microscopy (left panel), quantification, and statistical analysis were shown (right panel). (n = 3, one-way ANOVA). **F** hTERT-RPE-1 F3A3 cells were treated with indicated compounds for 24 h. Protein expression was analyzed by Western blot. (n = 3, one-way ANOVA). **G** hTERT-RPE-1 F3A3 cells were treated with indicated compounds for 24 h. After FerroOrange incubation, cells were then collected and measured by flow cytometry. (n = 3, one-way ANOVA). **H** Data are from one representative experiment as (**E**). hTERT-RPE-1 F3A3 cells were treated with indicated compounds for 24 h, followed by FerroOrange staining for microscopy (left panel), quantification and statistical analysis were shown (right panel). (n = 3, one-way ANOVA). **I** hTERT-RPE-1 F3A3 cells were pretreated with indicated compounds for 24 h, followed by an 8 h treatment with either a vehicle or 200 nM RSL3. Cells were then collected for flow cytometry. (n = 3, one-way ANOVA).

We observed the downregulation of transferrin receptor protein 1 (TFR1) but also ferritin (H chain) and ferroportin (FPN) upon FOXO3 activation (Fig. 5A), which indicates that regulation of iron homeostasis may contribute to FOXO3-mediated ferroptosis inhibition. Interestingly, a thymidine-induced cell cycle arrest was also accompanied by downregulation of TFR1, ferritin, and FPN expression, whereas a palbociclib-induced G1 arrest did not show any regulation of these proteins. FerroOrange is a fluorescent probe that can be used to detect intracellular labile iron [33] (Fig. 5B). Interestingly, FOXO3 activation led to a decreased basal level of cellular Fe^2+^(Fig. 5C). Addition of excessive Fe^2+^ or Fe^3+^ to the culture medium resulted in a concomitant increase of cellular Fe^2+^, which was lowered after FOXO3 activation (Fig. 5C), suggesting that FOXO3.A3 expression restricted iron uptake in these cells. Furthermore, consistent with the inhibition of ferroptosis by a thymidine block-induced arrest, we also observed a decrease in cellular Fe^2+^ following a thymidine block (Fig. 5D). This result was confirmed by fluorescent imaging **(**Fig. 5E).

To further confirm that FOXO3.A3-induced downregulation of TFR1 is functional in mediating ferroptosis resistance, we used a small molecule inhibitor for iron transport ferristatin II [34], which downregulates transferrin receptor 1 (TFR1) expression via receptor degradation (Fig. 5F). Treatment of hTERT-RPE-1 cells with ferristatin II decreased the cellular labile iron levels (Fig. 5G, H) and desensitized cells to ferroptosis (Fig. 5I). These results suggest that FOXO3 activation reduces hydroxyl radical formation through concomitantly increasing reductive capacity and decreasing cellular iron content. As we observed that palbociclib treatment did not reduce iron uptake and did not change ferroptosis sensitivity, despite the induction of G1 arrest and increased reductive capacity, we conclude that in hTERT-RPE-1 cells, a combination of redox and iron metabolism regulation is needed to regulate ferroptosis sensitivity. In case of FOXO3 activation, this regulation occurs on top of regulating expression of proteins like ACSL4 and PEX5, involved in lipid metabolism.

## Discussion

Here, we show that in the untransformed hTERT-RPE-1 cell line FOXO3 activation reduces sensitivity to ferroptosis. A combination of FOXO3-dependent regulation of lipid-, iron -and redox metabolism lowers sensitivity to ferroptosis, and this is linked to the ability of FOXOs to arrest cells in G1. However, a comparison of the FOXO3-dependent cell cycle arrest to cell cycle arrest caused by various other treatments showed that for hTERT-RPE-1 cells, the ability to induce a cell cycle arrest is not directly linked to ferroptosis protection. Cell cycle arrest induced by culturing cells in serum-free conditions or in the presence of 95°C heat-inactivated serum efficiently protected against ferroptosis induction by RSL3 treatment (Fig. 3D). We also observed that these treatments resulted in a reduction in cellular iron level (Fig. 5D). These observations are in agreement with Gao, et al. who showed that serum-free culturing of cells protects against ferroptosis because of a lack of transferrin, otherwise present in serum, which is required to import iron into cells [28].

Cell cycle inhibition by cell-cell contact also protected against ferroptosis, but in contrast to recent literature [27, 35] (reviewed in [36]), in our experimental setup, this was not dependent on E-cadherin/YAP signaling and ACSL4 downregulation. In most epithelial cell types, E-cadherin is the major cadherin responsible for forming and maintaining their epithelial phenotype [37]. However, it has been reported that hTERT-RPE-1 cells, in contrast to most epithelial cells, are different in terms of the major cadherin subtype (N-Cadherin) ([38] see also [39]), suggesting that signaling other than E-cad/YAP1 mediates density-dependent ferroptosis sensitivity.

Interestingly, FOXO and YAP1 have been shown to interact [40] and we observed FOXO3 activation to reduce (Fig. 3C) and YAP1 activation to increase ACSL4 expression (Fig. 3B). YAP1 activation of ACSL4 expression likely occurs through YAP1-TEAD interaction and therefore these observations suggest the possibility that following FOXO3 activation YAP1 switches from TEAD binding to FOXO3 binding and that this results in reduced ACSL4 expression. The possibility that FOXOs, through interaction with transcriptional coactivators, regulates transcriptional output of other transcription factors is already shown in case of the co-activator b-catenin, where under redox stress, b-catenin switches from binding to TCF to binding to FOXO3 (refs. [41, 42]). The possibility that YAP switching from TEAD to FOXO3 may regulate transcriptional output of both TEAD and FOXO3 is currently under investigation.

In contrast to a recent study [26], our results show that a palbociclib-induced G1 arrest in hTERT-RPE-1 cells did not result in reduced ferroptosis sensitivity. Lee et al. showed that cell cycle arrest of cancer cells, including arrest induced by palbociclib treatment, reduces ferroptosis sensitivity by cell cycle arrest-dependent lipid droplet formation [26].In agreement with this study, we also observed for hTERT-RPE-1 cells that a palbociclib treatment induced G1 arrest and increased lipid droplet formation. However, a FOXO3-dependent G1 arrest did not change, or at later time points even lowered lipid droplet formation. A lack of lipid droplet formation after FOXO3 activation agrees with previous studies that showed that loss of FOXO3 increases and FOXO3 activation reduces lipid droplet formation in different cell types and conditions [43]. Lipid droplet formation through the action of SIRT6 has also been proposed to regulate FOXO3 as part of a feed-back mechanism to connect cell cycle progression to lipid droplet formation (reviewed in [44]). Thus, our data suggest that a G1 cell cycle arrest per se is not linked to lipid droplet formation and that ferroptosis sensitivity is not solely determined by lipid droplet formation but requires concomitant changes in either lipid and/or redox metabolism. This is corroborated by the observation that a thymidine block does increase ferroptosis resistance and an increase in lipid droplet formation, but also affects TFR1 and ACSL4 expression, which we did not observe after palbociclib treatment.

Inhibition of cystine import and consequently lowering de novo GSH synthesis by erastin treatment induces ferroptosis. Initially, this was linked to the dependency of GPX4 function on glutathione, but we show that erastin treatment also impairs the ability to lower H_2_O_2_ levels. This suggests that regulation of H_2_O_2_ levels contributes to ferroptosis sensitivity. However, genetic disruption of GCLC does not seem to induce ferroptosis despite eliminating de novo GSH synthesis [45]. Furthermore, administration of cyst(e)inase [46], a drug that depletes cysteine and cystine, causes cell cycle arrest and death through ferroptosis in cancer cells due to depletion of intracellular GSH and ensuing elevated ROS. Importantly, and like genetic disruption of GCLC, this treatment results in no apparent toxicities in mice [47]. Combined, these observations may indicate that other cysteine-derived, sulfur-containing metabolites like hydropersulfides [48] can suppress ferroptosis, in parallel to GSH. Alternatively, the redox balance of most cancer cells contrasts to normal cells, where cancer cells produce increased levels of ROS, which is counterbalanced by increased reductive capacity to prevent ROS-induced cell death. Consequently, cancer cells will suffer from increased sensitivity to ferroptosis when reducing cystine/cysteine and ensuing GSH levels. Combined, this implicates that the level of endogenous ROS can be sufficient to induce ferroptosis when the reductive capacity is insufficient. This supports a role in ferroptosis protection for the observed FOXO3-induced increase in reductive capacity (Fig. 4C, D, Supplementary Fig. 2C).

Our results on FOXO3-mediated regulation of ferroptosis indicate that regulation of iron metabolism in this context is central, next to the regulation of reductive capacity. Extracellular Fe^3+^ binds transferrin and is transported into cells by the transferrin receptor (TFR1). Fe^3+^ is reduced to Fe^2+^ in endosomes, and this is released into the cytosol, generating the (free) labile iron pool. To prevent an excess buildup of free iron, Fe^2+^ is sequestered by ferritin. Otherwise, free Fe^2+^ can also be exported from cells through specific transporters such as SLC40A1. Dysregulation of abovementioned proteins can lead to an increase of the labile iron pool in the cytoplasm, and thereby enhances susceptibility to ferroptosis [49]. Fe^2+^ catalyzes the Fenton reaction to generate hydroxyl radicals from hydrogen peroxide. In addition, the lipid hydroperoxides formed can also react with iron to generate highly reactive lipid radicals, which may further contribute to ferroptosis [50]. In addition to controlling cellular reductive capacity, we show that FOXO3 activation regulates iron metabolism, likely by reducing TFR1 expression. This resulted in reduced iron uptake (Fig. 5C–E) and consequently reduced lipid peroxidation following FOXO3 activation (Fig. 1G, H). Using an inhibitor against TFR1, we confirmed that a reduction in TFR1 protects hTERT-RPE-1 cells from ferroptosis, indicating that TFR1 regulation by FOXO3 contributes to the regulation of ferroptosis by FOXO3. Interestingly, we also observe a concomitant downregulation of iron-binding protein ferritin (H chain), which could result in an increased labile iron pool and a consequent increased sensitivity to ferroptosis. However, as we observe a reduction in intracellular iron, this observation suggests that the level of ferritin is either still sufficient to minimize the labile iron pool or that ferritin expression is determined by iron import.

Taken together, our data show that after FOXO3 activation regulates ferroptosis sensitivity in a multilayered manner. First, hydroxyl radical formation through the Fenton reaction is apparently most efficiently inhibited by a combined increase in reductive capacity and a reduction in iron uptake and consequent lowering of cellular iron level. Cell cycle arrest resulting in increased reductive capacity without concomitant iron regulation does not result in efficient protection against ferroptosis. Regulation of lipid metabolism by increased lipid droplet formation appears less relevant in hTERT-RPE-1 cells, where downregulation of ACSL4 and, in case of FOXO3 activation, also PEX5, correlates with ferroptosis protection. This shows that FOXO3 activation acts to protect arrested cells from ferroptosis and generalizes the function of FOXO3 to mediate cell survival under a variety of different stress conditions.

## Methods

### Cell culture

hTERT-immortalized Retinal Pigment Epithelial cells (HTERT-RPE-1, female origin) were cultured in DMEM F12 medium containing 10% (v/v) fetal bovine serum and 1% (v/v) penicillin/streptomycin and 2 mM L-glutamine. 293T Human Embryonic Kidney cells (HEK293T, fetal origin) used for lentiviral production and the human osteosarcoma U2OS cells were cultured in DMEM-High Glucose medium containing 10% (v/v) fetal bovine serum, and 1% (v/v) penicillin/streptomycin, and 2mM L-glutamine. Cells were grown at 37 °C under 6% CO_2_ atmosphere. Cells were split and the medium refreshed twice weekly, with routine mycoplasma tests confirming negativity. A treatment with 100 nM doxycycline for 24 h was used to activate FOXO3 expression in F3A3 cells. For cell cycle arrest, cells were pretreated 24 h with 500 nM Palbociclib, 2.5 mM Thymidine, 200 nM Nocodazole, serum-free conditions, or 95 °C heat-inactivated serum. For experimental setups, cells were counted using a Countess automated cell counter (Invitrogen) using trypan blue.

### Cell lines

HTERT-RPE-1, U2OS, and HEK293T cells were obtained from the ATCC. Transfection of third-generation packaging vectors using Polyethylenimine into HEK293T cells generated lentiviral particles. Generation of RPE FUCCI cells was previously described [51]. Lentiviral cDNA expression vectors expressing FOXO3.A3 were generated using Gateway cloning in the pINDUCER20 [52] (Addgene #44012) doxycycline-inducible expression system [53], followed by selection with 400 μg/mL geneticin for 2 weeks. After selection, single cells were sorted into 96-well plates and then expanded to create monoclonal HTERT-RPE-1 FOXO3.A3 (HTERT-RPE-1 F3A3) lines. The inducible expression of FOXO3 was confirmed by Western blotting, and cells with high induced FOXO3 expression were sorted for subsequent experiments. Generation of hTERT-RPE-1 pTON-EGFP-FOXO3 cells and U2OS pTON-EGFP-FOXO1 cells was performed as described previously [54].

Plasmids containing the genetic sequence for NLS-HyPer7 and NES-HyPer7 were a kind gift from Dr. Vsevolod Belousov [54]. Generation of NLS-HyPer7 and NES-HyPer7 lentiviruses, infection of RPE F3A3 cells was performed as described previously [32]. Single cells with high Hyper7 expression were sorted into a 96-well plate by using FACS, then expanded to create monoclonal lines.

The pInducer20-FLAG-YAP1 S5A plasmid, a kind gift from the Gloerich lab [29], was used to express mutant YAP1 in HTERT-RPE-1 cells. Confirmation of expression was done after addition of doxycycline using Western blot for YAP1.

### Cell seeding density and ferroptosis induction

Cells were seeded in 6-well plates for subsequent Western blot, quantitative real-time PCR, and flow cytometry experiments. For live cell imaging, cells were seeded in 8-well chamber slides (Ibidi). The cell seeding density varied across experimental groups. Briefly, for 6-well plates, in the sparse group, cells were seeded at 3 × 10^4^ cells per well; in the dense group, at 3 × 10^5^ cells per well; and in the standard experimental group, at 6 × 10^4^ cells per well. For 8-well chamber slides, cells were seeded at 8 × 10^3^ cells per well.

Ferroptosis was induced with RSL3 or erastin the day after cell plating or pre-treatments with a vehicle, doxycycline, or other required treatments, depending on the experiment. Liproxstatin-1 or ferrostatin-1 was added to the medium at the same time as RSL3 for rescue of ferroptosis.

### Protein lysates and Western blot

Protein lysates were obtained by washing cells with PBS and subsequently lysed and scraped in 1x Sample Buffer (2% SDS, 5% 2-mercaptoethanol, 10% glycerol, 0.002% bromophenol blue, 300 mM Tris-HCI, pH 6.8). After heat denaturation at 95 °C, 20–40 μg of proteins were run in SDS-PAGE and transferred to Immobilon PolyScreen PVDF transfer membranes. Samples were blocked for 1 h at 4 °C in TBS-Tween (1% v/v), containing 2% BSA. Primary antibodies (Vinculin 1:10 000, FOXO3 1:5 000, p27 1:2 000, SOD2 1:2 000, BCL6 1:1 000, ACSL4 1:2 000, TFR1 1:2 000, YAP1 1:2 000, PEX5 1:5 000, Ferritin 1:1 000, FPN 1:2 000, SLC7A11 1:1 000, GPX4 1:500, pAkt/PKB Ser473 1:2 000, pFOXO1 Thr24/FOXO3a Thr32 1:2 000) were applied in TBS-Tween 0.1% at 4 C overnight followed by 1 h RT incubation of secondary HRP-conjugated antibodies (1:10 000) targeting either mouse or rabbit IgG in TBS-Tween 0.1%. ACSL4 siRNA (Horizon Discovery) was used to determine band height of ACSL4. Scrambled siRNA was used as control. Protein abundance was then detected by enhanced chemiluminescence method (ECL) using ImageQuant LAS (GE HealthCare) with ImageQuant LAS4000 (version 1.3) software. Contrast was adjusted for clarity by linear image processing in Adobe Photoshop if required. Statistical analysis from three independent experiments is shown in the right panels. The expression levels of the target protein were quantified relative to the control group, which was set to 1. Full-length and uncropped Western blot images corresponding to all figures presented in this study are included in the Supplementary Material.

### RNA extraction and real-time PCR

RNA purification was performed with the RNeasy Mini Kit with DNase treatment (Qiagen), following the manufacturer’s protocol. DNase-treated RNA was used for cDNA synthesis using iScript cDNA synthesis kit (Bio-Rad). Afterward, cDNA was subjected to qPCR using FastStart Universal SYBR Green Master mix (Roche) with the CFX Connect Real-time PCR system (Bio-Rad). Target genes were amplified using specific primer pairs (Supplementary Table 1). The relative expression of individual genes, compared to expression levels under control conditions, was determined using the 2^−ΔΔCt^ method from Ct readouts for each gene, normalized to β-Actin (Table 1).

**Table 1:** Materials used in this study.

Reagent or resource	Source	Identifier
Antibodies		
Vinculin	Sigma-Aldrich	V9131
FOXO3	Cell Signaling Technology	2497
P27	BD Biosciences	610241
SOD2	Enzo Life Sciences	ADI-SOD-110
BCL6	Abcam	Ab183308
ACSL4	Thermo Fisher	PA5-27137
TFR1	Thermo Fisher	13-6890
YAP1	Santa Cruz	sc-101199
PEX5	Thermo Fisher	CF501430
Ferritin Heavy Chain	Abcam	Ab16875
FPN	Thermo Fisher	PA5-22993
SLC7A11	Cell Signaling Technology	12691
GPX4	Santa Cruz	sc-166570
pFOXO1 (Thr24)/FOXO3a (Thr32)	Cell Signaling Technology	9464
pAkt/PKB (Ser473)	Cell Signaling Technology	4060
Goat Anti-Rabbit IgG (H + L)-HRP	Bio-Rad	170-6515
Goat Anti-Mouse IgG (H + L)-HRP	Bio-Rad	170-6516
Hoechst33342	Thermo Fisher	62249
DAPI	Sigma-Aldrich	D9564
Chemicals, peptides, and recombinant proteins		
DMEM F12	Sigma-Aldrich	D8062
Fetal Bovine Serum (FBS)	BODINCO BV	S00KL10004
Glutamine	Lonza	17-605E
Geneticin (G418)	Thermo Fisher	11811064
Penicillin/Streptomycin	Sigma-Aldrich	P0781
Trypsin	Sigma-Aldrich	T3924
RSL3	Cayman Chemical	19288
Erastin	Bio-Connect	S7242
PKB inhibitor VIII	Chemcruz	SC-202048A
GSH	Sigma-Aldrich	PHR1359
GSSG	Sigma-Aldrich	G4376
MDA	Sigma-Aldrich	63287
TEP-D2	Santa Cruz	sc-208708
Ferrostatin-1	Sigma-Aldrich	SML0583
Liproxstatin-1	Sigma-Aldrich	SML1414
Doxycycline	Sigma-Aldrich	D9891
BSA	Sigma-Aldrich	A9647
H_2_O_2_ solution	Sigma-Aldrich	H1009
Ammonium iron (II) sulfate hexahydrate	Sigma-Aldrich	09719
Ammonium iron (III) citrate	Sigma-Aldrich	F5879
Palbociclib	Sigma-Aldrich	PZ0383
Thymidine	Sigma-Aldrich	T1895
Nocodazole	Sigma-Aldrich	M1404
BODIPY^TM^ 581/591 C11	Thermo Fisher	D3861
BODIPY^TM^ 493/503	Thermo Fisher	D3922
FerroOrange Live Cell Dye	Sigma-Aldrich	SCT210
Ferristatin II	Cayman Chemical	36621
Experimental models: Cell lines		
HEK293T		
hTERT -RPE-1 F3A3		
hTERT -RPE-1 F3A3 FUCCI		
hTERT -RPE-1 F3A3 p27-/- FUCCI		
hTERT -RPE-1 NLS-HyPer7 F3A3		
hTERT -RPE-1 NES-HyPer7 F3A3		
hTERT -RPE-1 YAP1 S5A		
hTERT-RPE-1 pTON-EGFP-FOXO3		
U2OS pTON-EGFP-FOXO1		
Oligonucleotides		
ACSL4 siRNA	Horizon Discovery	L-009364-00-0005
RT-PCR primers	IDT	Listed in Supplementary Table 1
Software and algorithms		
GraphPad Prism 10	Adobe	https://www.graphpad.com/ scientific-software/prism/
Fiji	ImageJ	https://imagej.net/Fiji
Stardist ImageJ plugin	Schmidt et al. [56].	https://github.com/stardist/ stardist-imagej.git
Adobe Illustrator	N/A	https://www.adobe.com/nl/ products/ illustrator.html
Adobe Photoshop 2022	N/A	https://www.adobe.com/nl/ products/ photoshop.html

### Flow cytometry

Cells cultures subjected to different treatments were collected (including floating dead cells) in 200 μL ice-cold PBS/DAPI solution and dissociated into single cells prior to flow cytometry analysis (BD FACS Celesta #660345). At least 1 × 10^4^ cells were analyzed with BD FacsDiva Software.

Cell viability was analyzed using 1 μg/mL DAPI staining. The percentage of DAPI-positive dead cells was determined using the BD FACS Celesta with a 405 nm laser. Cell cycle profile was analyzed by gating live cells using FUCCI cell cycle reporter as described previously [54].

Lipid peroxidation was examined using C11-BODIPY^581/591^ staining as described previously [55]. The following day after cell seeding, cells were pretreated with a vehicle or doxycycline for 24 h. Then, cells were incubated with 1.5 µM C11-BODIPY^581/591^ for 30 min at 37 °C, followed by lipid peroxidation induced with H_2_O_2_ or ferroptosis inducers for 5 h. Lipid peroxidation in these cells was assessed using both the 488 nm and 561 nm lasers to detect the oxidized and reduced signals of C11-BODIPY^581/591^, respectively. The 561_ex_/488_ex_ ratio was used as an indicator of lipid peroxidation levels in these cells. For each group, the ratio was normalized to the vehicle condition.

### Live imaging of cell viability

Cells with FUCCI cell cycle reporter were plated on 8-well Ibidi slides 1 day before the start of the time-lapse experiments for 24 h. The treatment with RSL3 or Erastin was added just prior to imaging. Imaging was performed using a Zeiss Cell Observer microscope (Zeiss Microsystems). Images were taken every 20 min. Total cell populations were visualized based on FUCCI signals. Dead cells were highlighted using 1 μg/mL DAPI staining. Counting of cells was done using Fiji. An automatic threshold was set on unedited images, single pixels were deleted, and watershed was performed to split touching cells into two separate cells. Nuclei were segmented based on FUCCI signals (total cells) or DAPI-positive signals (dead cells) using the StarDist plugin [56]. Every three consecutive frames were analyzed together, and the mean value was used to determine the cell viability at that time point.

### Determination of lipid droplets

Lipid droplet was examined using BODIPY 493/503 staining as described previously [57]. The following day after cell seeding, cells were pretreated with a vehicle, doxycycline, or cell cycle inhibitor for 24 h or 48 h. Cells were then incubated with 2 µM BODIPY 493/503 for 15 min at 37 °C. Cells not treated with BODIPY 493/503 served as the negative control (NC). Lipid droplet levels were measured using the BD FACS Celesta with a 488 nm laser. Live cell imaging of lipid droplet was visualized using a SP8 confocal microscope (Leica Microsystems) with a 40× oil-water immersion objective. 2 µg/ml Hoechst33342 was used for staining the nuclei. Hoechst was excited at 405 nm and emission measured at 415–481 nm, BODIPY 493/503 was excited at 493 nm and emission measured at 500–520 nm. Lipid droplet fluorescence intensity per cell was quantified using Fiji software after background subtraction. At least 30 cells were analyzed. Z-stacks were acquired and displayed as maximum projections. All quantitative analyses were performed on unprocessed raw images.

### HyPer7 measurements

Monoclonal hTERT-RPE-1 F3A3 cells stably expressing either HyPer7-NLS or HyPer7-NES were plated in 8-well chamber slides (Ibidi). Measurements were performed on a Zeiss Cell Observer microscope (Zeiss Microsystems) using a 10× magnification and ZEN 2.6 blue software (v.2.6.76.00000). Cells were excited at 385 nm and 475 nm, and emission was measured using a 514/44 BP bandpass filter every 2 min. 50 µM H_2_O_2_ was added after measuring the first 10 min, upon which imaging was continued for another 120 min. For measuring HyPer7 oxidation by live imaging, we refer to the method described previously [58]. The 475_ex_/385_ex_ ratio was transformed into a percentage of HyPer7 oxidation by setting the lowest ratio measured to 0% and the highest ratio measured after exogenous H_2_O_2_ addition to 100%. The percentage of HyPer7 oxidation for each measurement was calculated using the following equation: HyPer7 oxidation (%) = (R_measured_−R_min_)/(R_max_−R_min_) × 100.

### Determination of intracellular iron content

Intracellular Fe^2+^ was examined using BioTracker^TM^ FerroOrange Live Cell Dye as described previously [59]. Briefly, the following day after cell seeding, cells were pretreated with a vehicle, doxycycline, or cell cycle inhibitor for 24 h or 48 h. 100 µM Fe^2+^/Fe^3+^ was added to the culture medium as a positive control. Cells were then incubated with 1 µM FerroOrange in PBS for 30 min at 37 °C. Cells not treated with FerroOrange served as the negative control (NC). Intracellular Fe^2+^ levels were measured using the BD FACS Celesta with a 488 nm laser. Live cell imaging of intracellular iron was visualized using a SP8 confocal microscope (Leica Microsystems) with a 40x oil-water immersion objective. 2 µg/ml Hoechst33342 was used for staining the nuclei. Hoechst was excited at 405 nm and emission measured at 415–481 nm, FerroOrange was excited at 542 nm and emission measured at 560–600 nm.

Intracellular iron fluorescence intensity per cell was quantified using Fiji software after background subtraction. At least 30 cells were analyzed. Z-stacks were acquired and displayed as sum projections. All quantitative analyses were performed on unprocessed raw images. Brightness and contrast were linearly adjusted for visualization purposes without altering the pixel intensity distribution.

### Determination of FOXO translocation upon ferroptosis induction

To monitor translocation of FOXO1/3 proteins during ferroptosis, cells stably expressing either GFP-tagged FOXO1 or GFP-tagged FOXO3 were plated in 8-well chamber slides (Ibidi). A treatment with 100 nM doxycycline for 48 h was used to activate FOXO1/3 expression in cells prior to imaging. 2 µg/ml Hoechst33342 was used for staining the nuclei. Subsequently, ferroptosis was induced by adding RSL3 or erastin, upon which the time-lapse imaging was performed every 15 min for a total of 6 h on a Zeiss Cell Observer microscope (Zeiss Microsystems) using a 10× magnification and ZEN 2.6 blue software (v.2.6.76.00000). Cells were excited at 385 nm and 475 nm and emission was measured using a 514/44 BP bandpass filter. The treatment of PKB inhibitor VIII was used as a positive control for FOXO1/3 nuclear translocation. Images were analyzed using Fiji software, and the fluorescence intensity of GFP-FOXO1/3 in the nucleus versus the cytoplasm was quantified to assess FOXO1/3 nuclear translocation upon ferroptosis induction.

### LC-MS analysis of GSH and MDA

Samples were transferred to 2 mL Eppendorf safe lock tubes, homogenized using stainless steel beads (0.9-2 mm) for 2 × 2 min at 4 °C in a Bullet Blender (Next Advance, Troy, NY, USA), and evaporated to dryness in a Labconco Centrivap (VWR, Amsterdam, The Netherlands). For GSH / GSSG analysis, 300 µL Mili-Q water, 300 µL methanol, and 450 µL chloroform (all organic solvents were ULC-MS grade and purchased from Biosolve) were added, and the samples were incubated for 2 h in a VWR thermostated shaker (900 rpm, 37 °C). After centrifugation at room temperature (10 min, 15,000 × *g*), the upper aqueous phase was quantitatively transferred to a clean 1.5 µL Eppendorf tube and evaporated to dryness overnight in a Labconco Centrivap. The residue was dissolved in 100 µL Mili-Q water and transferred to an injection vial for LC-MS analysis.

The LC-MS analysis was performed using a 2.1 × 100 mm HSS T3 C18 column (2.1 × 100, 1.8 µm) connected to a related VanGuard column, both purchased from Waters (Etten-Leur, The Netherlands). The column was installed into an Ultimate 3000 LC system. The column outlet was coupled to a Q-Exactive FT mass spectrometer equipped with a HESI ion source. The UPLC system was operated at a flow rate of 350 μL min^−1^, and the column was kept at 30 °C. The mobile phases consisted of 0.1% formic acid in water (A) and acetonitrile (B), respectively. Upon 5 µL sample injection, the system was kept at 0% B for 1.5 min followed by a 6 min linear gradient of 0-30% B. Thereafter, the gradient increased linearly to 70% in 4 min and kept at 70% for 0.5 min followed by column regeneration at 0%B for 4 min.

For MDA analysis, an adapted protocol of Kartavenka et al. [60] was used. In short: the homogenized, evaporated samples were dissolved in 200 µL methanol and centrifuged for 10 min at 10 °C and 17,000 × *g*. A volume of 180 µL was transferred to clean 1.5 mL Eppendorf vials and evaporated to dryness. Sample residues were dissolved in 20 µL Milli Q water and 30 µL methanol. After addition of 10 µL 5 µM TEP-D2 (internal standard) and 10 µL 2.1 mM dansyl chloride in 750 mM HCl in Milli Q water, the samples were incubated for 2 h at 45 °C with shaking at 300 rpm. After cooling the samples on ice for 5 min, 10 µL 4 M ammonium hydroxide was added to neutralize the pH of the sample. The samples were vortexed mixed, centrifuged for 2 min at 10 °C and 17,000 × *g*, transferred to injection vials and subjected to RP analysis on a Waters HSS T3 column (2.1 × 100 cm, 1.8 µm) using a gradient as described earlier [60].

Mass spectrometry data were acquired on an Exploris 480 MS coupled to a Vanquish LC over a scan range of m/z 60 to 900. The system was operated at 140,000 mass resolution and at −2.5 kV for GSH/GSSG analysis and 3 kV for MDA analysis, respectively. Further source settings were: transfer tube and vaporizer temperature 350 °C and 300 °C, and sheath gas and auxiliary gas pressure at 35 and 10, respectively. For high mass accuracy, mass calibration was performed before each experiment. The Raw data files were processed and analyzed using XCalibur Quan software.

### Statistics and reproducibility

All cell culture experiments were conducted independently at least three times with similar results. Technical replicates were evaluated for consistency, and experiments were repeated if the variation among replicates exceeded acceptable thresholds. Investigators were not blinded during sample processing; however, for outcome assessment, data were processed and analyzed without reference to group labels to minimize bias. Statistical analysis for image analysis, flow cytometry, and immunoblot results was performed by using Graphpad Prism 10. Comparisons between two groups were performed using unpaired t-tests; for multiple groups, one-way ANOVA was applied. Details on the biological replicate sample sizes (n = x), type of test, and exact p-values are indicated in the figure legends. The data are presented as mean ± standard deviations (SD). A p-value < 0.05 was considered significant and indicated above the horizontal bars in figures. All findings reported here were observed independently at least three times, as stated in the figure legends.

## Supplementary information

Supplementary Figs. 1 and 2, legends, and flow cytometry gating strategies

Full-length and uncropped blots

Overview of RT-PCR primers used for this study

## Data Availability

Upon reasonable request, plasmids generated and additional information required to reanalyze the data reported in this study can be provided via the lead contact.

## Declaration of large language models in the writing process

In accordance with authorship and academic integrity guidelines, no large language models were used in the preparation of this work. The authors take full responsibility for the content of the publication.
