# Supplementary material for: Activation of a FOXO3-induced cell cycle arrest regulates ferroptosis
Source: Cell Death Discov. 2025 Oct 16;11:465. doi: 10.1038/s41420-025-02760-x (PMC12533257; doi:10.1038/s41420-025-02760-x)
Supplement: Supplementary file 1 — Supplementary Figs. 1 and 2, legends, and flow cytometry gating strategies [file 41420_2025_2760_MOESM1_ESM.pdf]

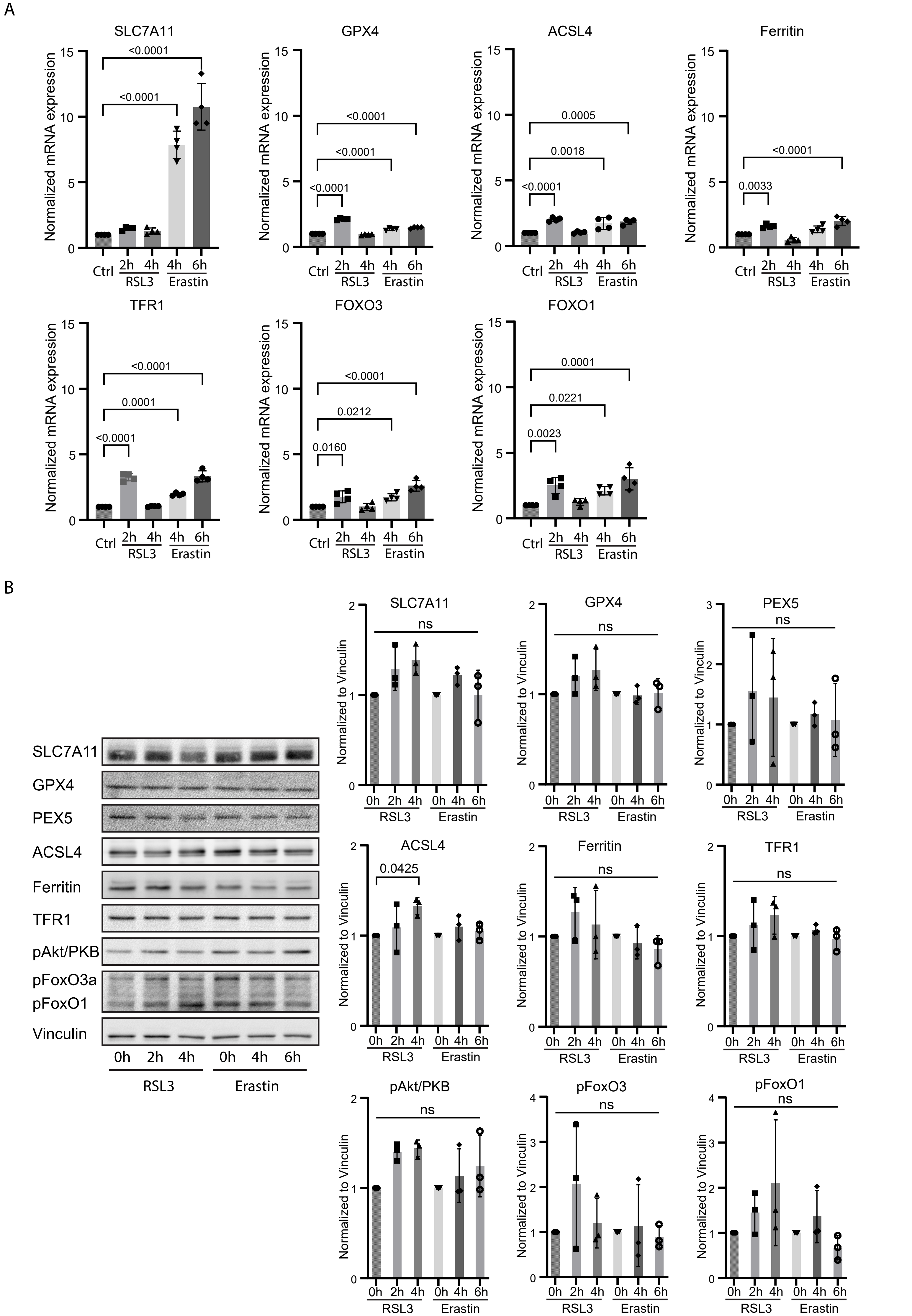
A
SLC7A11
GPX4
ACSL4
Ferritin
TFR1
FOXO3
FOXO1
Normalized mRNA expression
<0.0001
0.0005
0.0018
0.0033
0.0001
0.0212
0.0160
0.0221
0.0023
Ctrl
2h
4h
6h
RSL3
Erastin
B
SLC7A11
GPX4
PEX5
ACSL4
Ferritin
TFR1
pAkt/PKB
pFoxO3a
pFoxO1
Vinculin
0h
2h
4h
6h
RSL3
Erastin
pFoxO3
Normalized to Vinculin
ns
0.0425

C

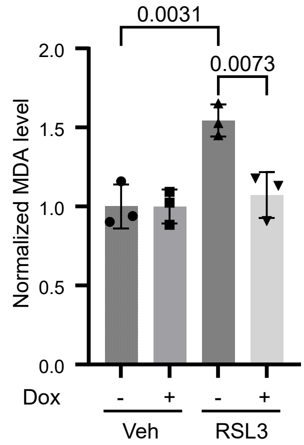
0.0031
0.0073
Normalized MDA level
2.0
1.5
1.0
0.5
0.0
Dox
-
+
-
+
Veh
RSL3

D

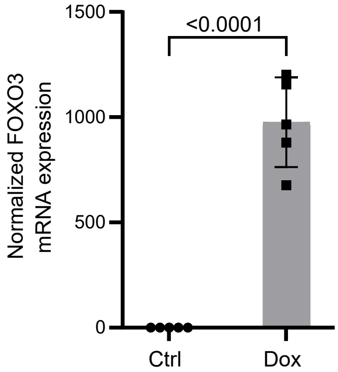
<0.0001
Normalized FOXO3 mRNA expression
1500
1000
500
0
Ctrl
Dox

E

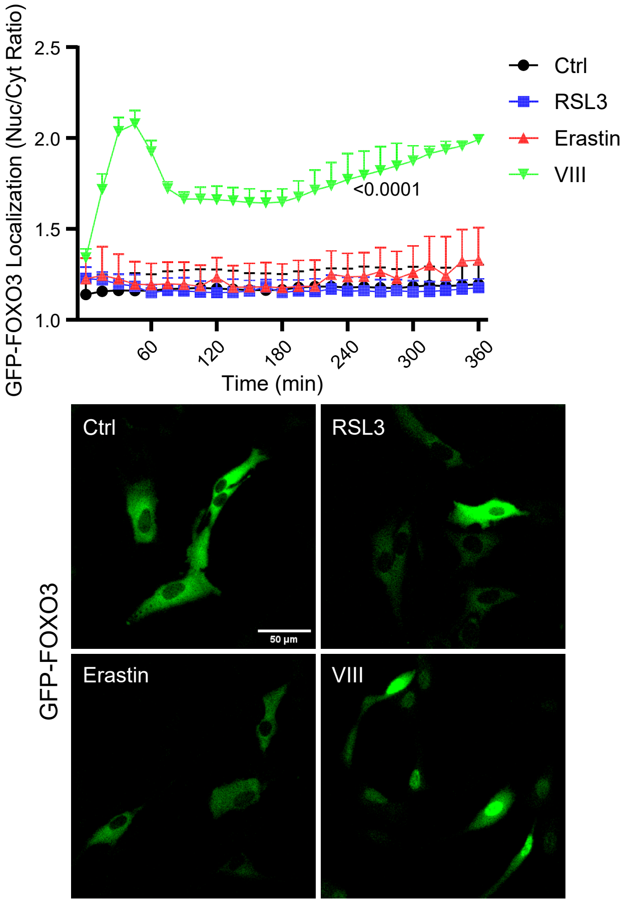
GFP-FOXO3 Localization (Nuc/Cyt Ratio)
2.5
2.0
1.5
1.0
<0.0001
60
120
180
240
300
360
Time (min)
Ctrl
RSL3
Erastin
VIII
GFP-FOXO3
50 µm

F

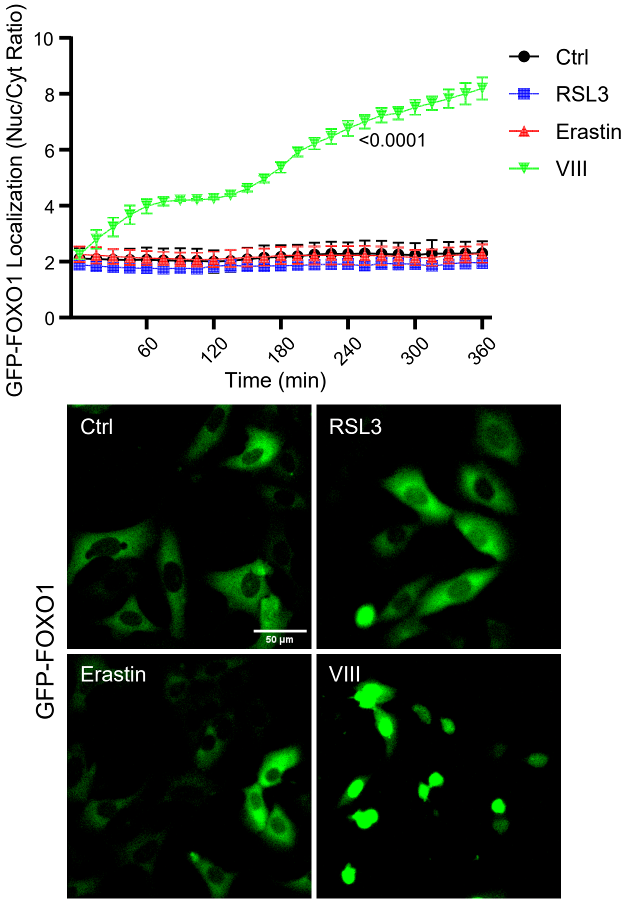
GFP-FOXO1 Localization (Nuc/Cyt Ratio)
10
8
6
4
2
0
<0.0001
60
120
180
240
300
360
Time (min)
Ctrl
RSL3
Erastin
VIII
GFP-FOXO1
50 µm

Supplementary Figure 1.

A. hTERT-RPE-1 cells were either left untreated or treated with indicated compounds (50 nm RSL3, 1 µM erastin), then collected for real-time PCR. The mRNA expression of target genes was normalized to β-Actin. (n=4, one-way ANOVA)

B. hTERT-RPE-1 cells were either left untreated or treated with indicated compounds (50 nm RSL3, 1 µM erastin), protein expression was analyzed by western blot. (n=3, one-way ANOVA)

C. LC-MS analysis of cellular MDA levels. hTERT-RPE-1 NLS-HyPer7 F3A3 cells were either left untreated or treated with doxycycline for 24 h, followed by the treatment of indicated compounds (50 nM RSL3 for 4 h). Then samples were collected for LC-MS analysis. (n=3, one-way ANOVA)

D. hTERT-RPE-1 F3A3 cells were either left untreated or treated with doxycycline for 24 h. Cells were then collected for real-time PCR. The mRNA expression of FOXO3 was normalized to β-Actin. (n=3, unpaired t test)

E and **F**. hTERT-RPE-1 pTON-EGFP-FOXO3 (**E**) and U2OS pTON-EGFP-FOXO1 (**F**) cells were left untreated (Ctrl) or treated with indicated compounds (50 nm RSL3, 1 µM erastin, 1 µM PKB inhibitor VIII), the fluorescence intensity of GFP-FOXO1/3 in the nucleus and cytoplasm was measured in time. Data are from one representative experiment, the fluorescence image corresponds to the time point 17 indicated in the graph above. (n=3, one-way ANOVA with significant differences from time point 17)

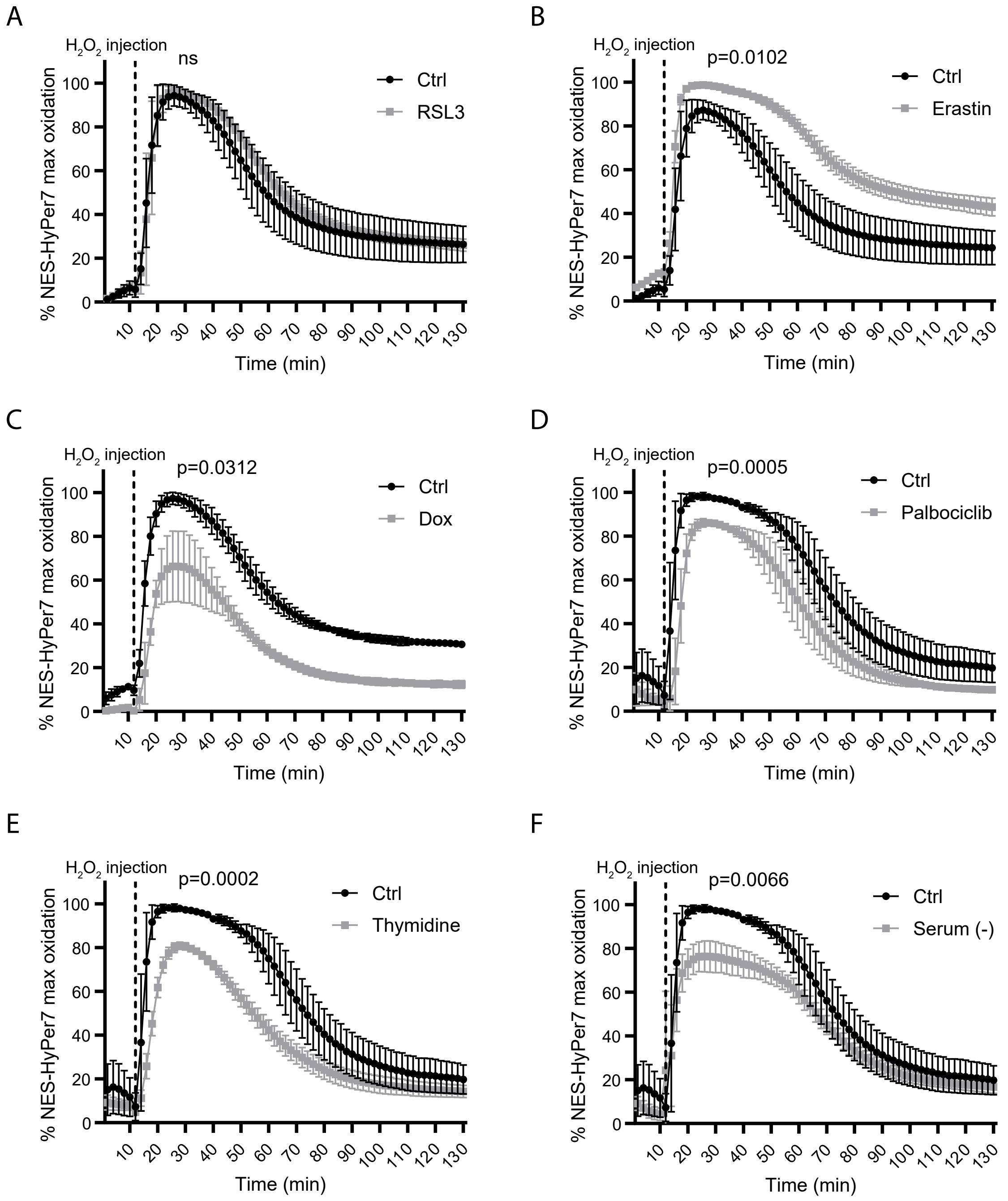
A
H_2O_2 injection
ns
Ctrl
RSL3
B
H_2O_2 injection
p=0.0102
Ctrl
Erastin
C
H_2O_2 injection
p=0.0312
Ctrl
Dox
D
H_2O_2 injection
p=0.0005
Ctrl
Palbociclib
E
H_2O_2 injection
p=0.0002
Ctrl
Thymidine
F
H_2O_2 injection
p=0.0066
Ctrl
Serum (-)
% NES-HyPer7 max oxidation
Time (min)

Supplementary Figure 2.

A-F. Same as Figure 4 A, B, D-G, hTERT-RPE-1 NES-HyPer7 F3A3 cells were used to confirm the effect of G1 cell cycle arrest on cellular reductive capacity across cell lines. In A and B, data are from one representative experiment; in D-F, data are from one representative experiment; the traces are deconvoluted for clarity. (Serum (-): serum deprivation). (n=3, unpaired t test with significant differences from time point 14)

Supplementary material 1: Flow Cytometry gating strategies

1. Cell viability

Example: Fig 1B

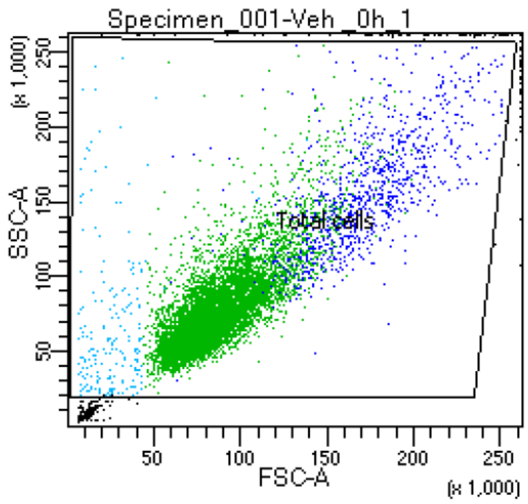

1, All cells were gated.

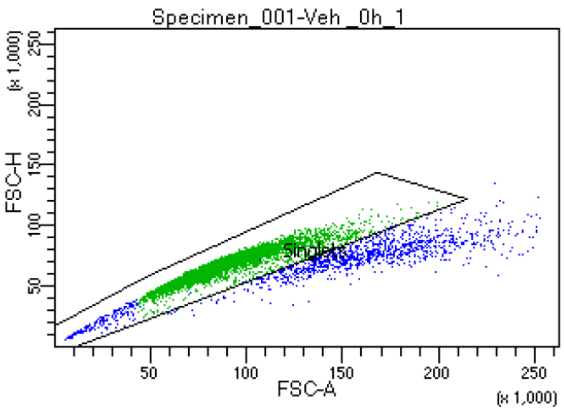

2, Single cells were gated.

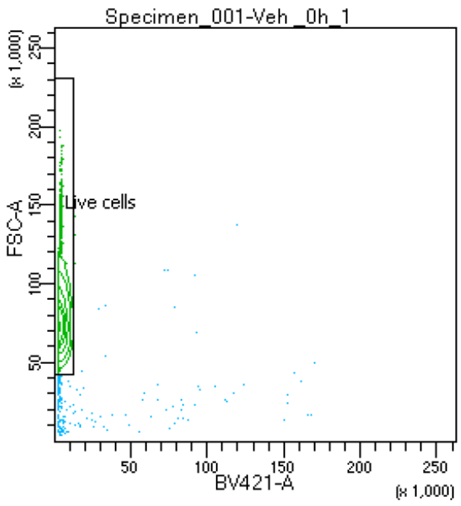

3, DAPI-negative cells were identified as live cells.

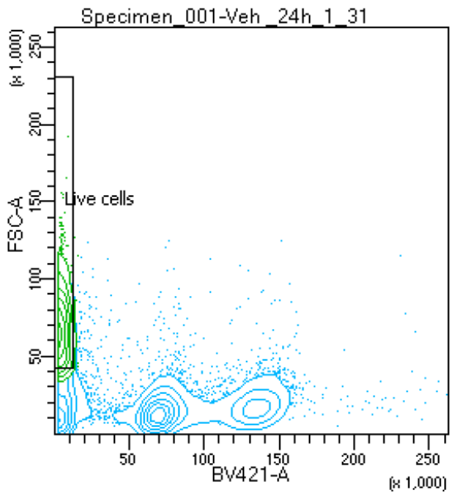

4, After treated with 50 nM RSL3 for 24 h, most of cells are DAPI-positive.

2. Cell cycle phase FUCCI cells

Example: Fig 1D

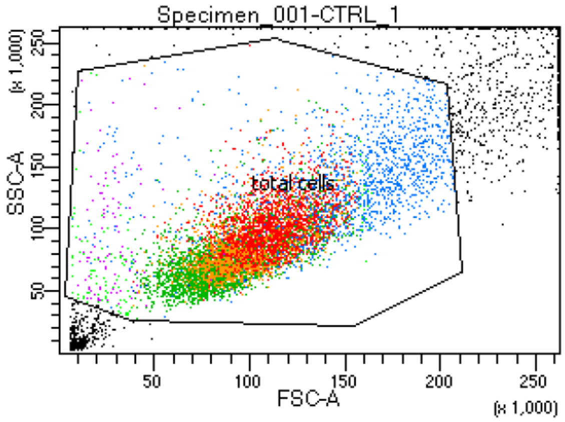

1, All cells are gated.

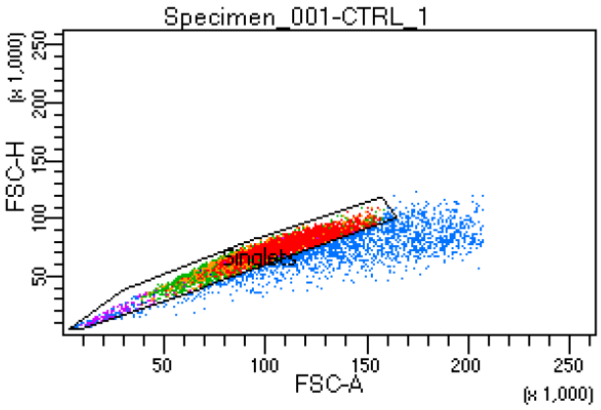

2, Single cells are gated.

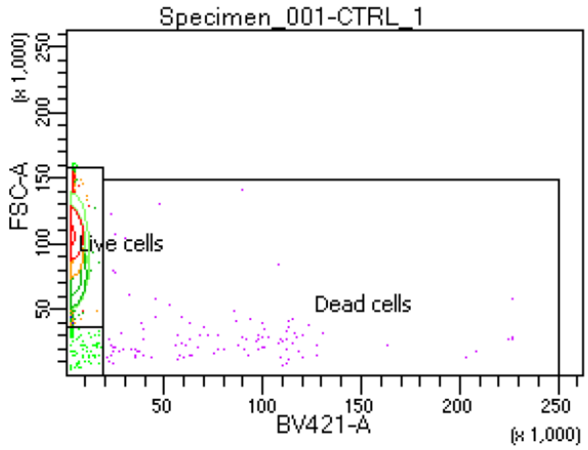

3, DAPI-negative cells were identified as live cells.

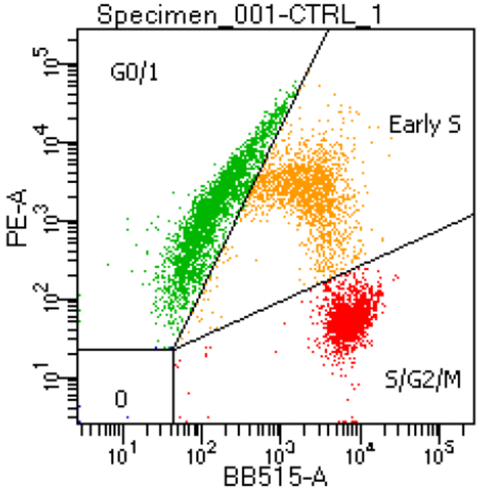

4, mKO2(+)mAG(-) cells (PE^{hi} $BB515^{low}$) were classified as G1phase, mKO2(+)mAG(+) cells (PE^{hi} $BB515^{hi}$) were classified as early S phase, and mKO2(-)mAG(+) cells (PE^{low} $BB515^{hi}$) were classified as late S/G2/M phase.

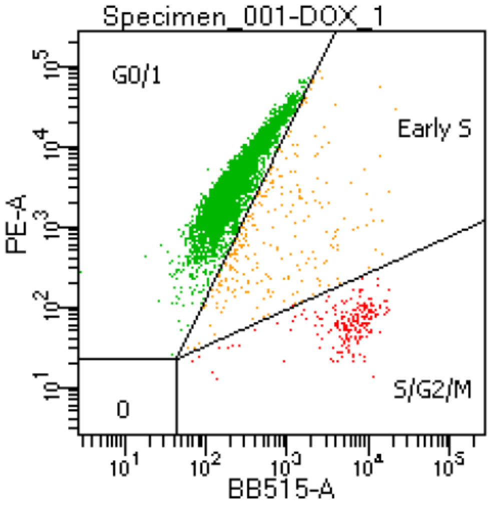

5, After treated with doxycycline for 24 h, more cells were arrested in G1 phase

3. C11-BODIPY$^{581/591}$

Example: Fig 1H

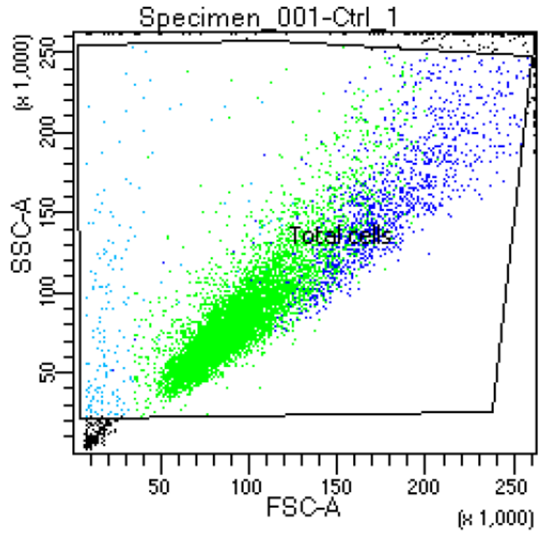

1, All cells are gated.

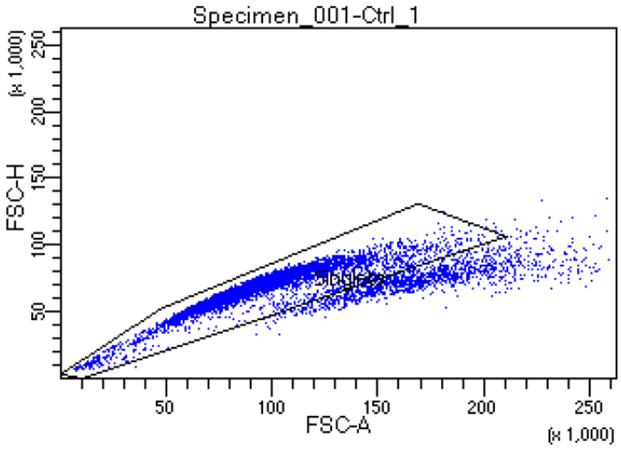

2, Single cells are gated.

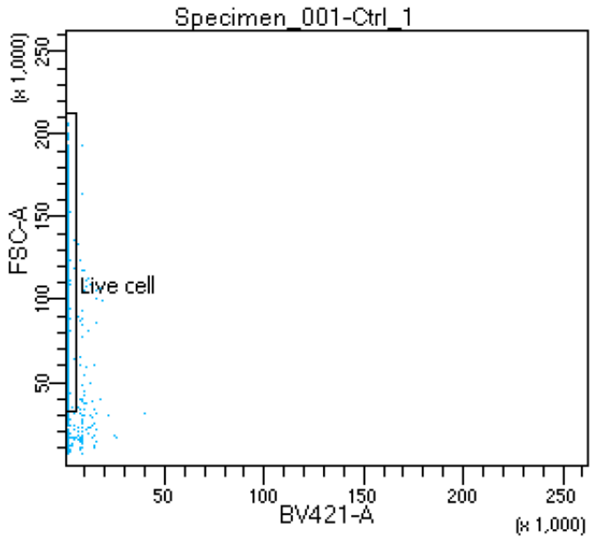

3, DAPI-negative cells were identified as live cells.

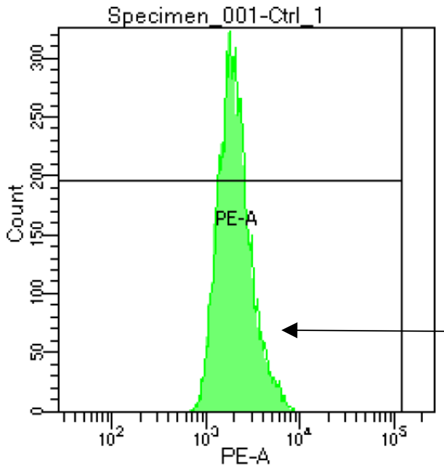

4, Reduced BODIPY C11 predominantly emits red fluorescence at 590 nm.

Mean intensity: 2066

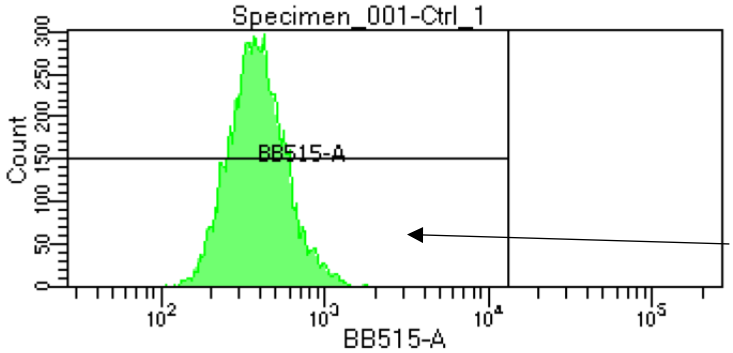

5, Oxidized BODIPY C11 predominantly emits green fluorescence at 510 nm.

Mean intensity: 403

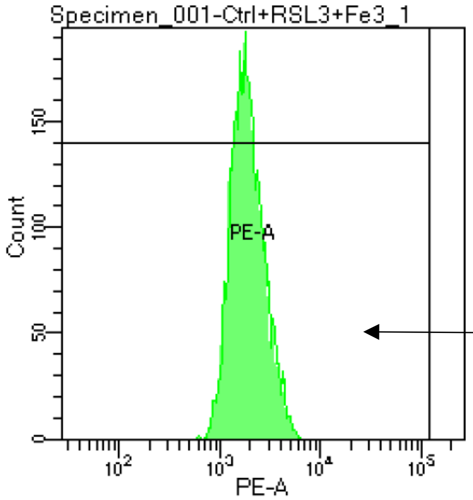

6, After treated with RSL3 and Fe^{3+} for 5 h, less reduced BODIPY C11 was detected.

Mean intensity: 1890

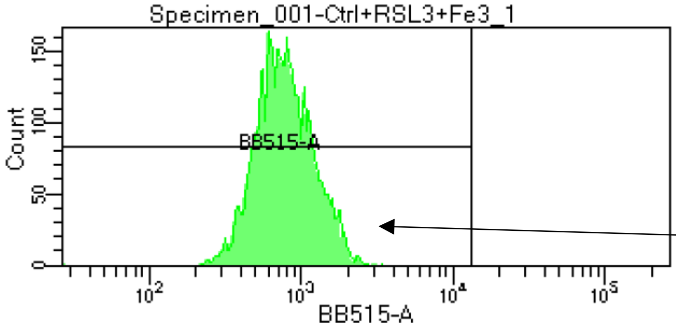

7, In addition, an increase in oxidized BODIPY C11 was observed.

Mean intensity: 817

4. Lipid droplets determination

Example: Fig 3E

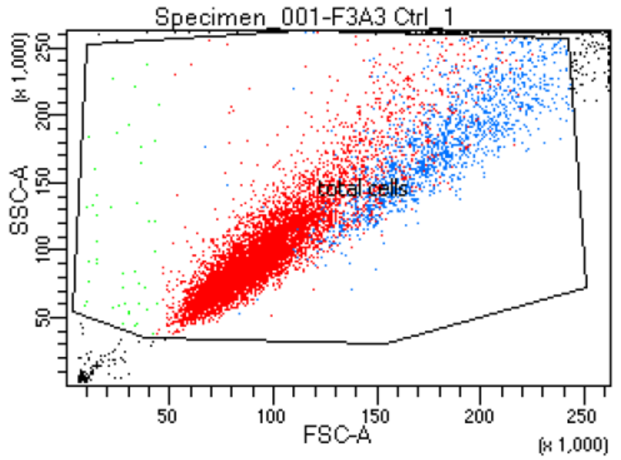

1, All cells are gated.

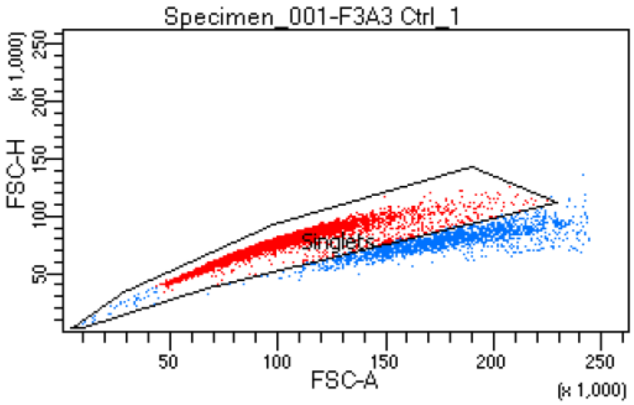

2, Single cells are gated.

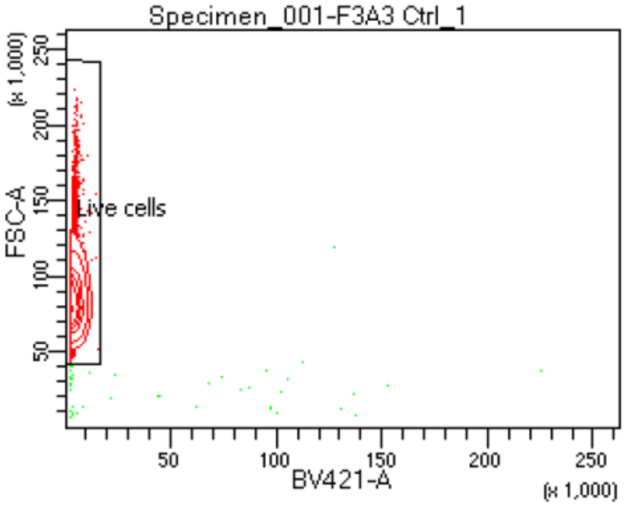

3, DAPI-negative cells were identified as live cells.

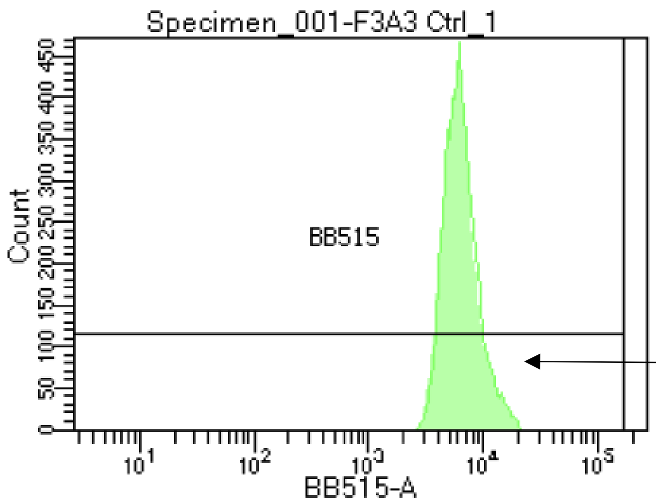

4, Lipid droplets were stained with BODIPY 493/503 and analyzed with the BB515 detector.

Mean intensity: 6409

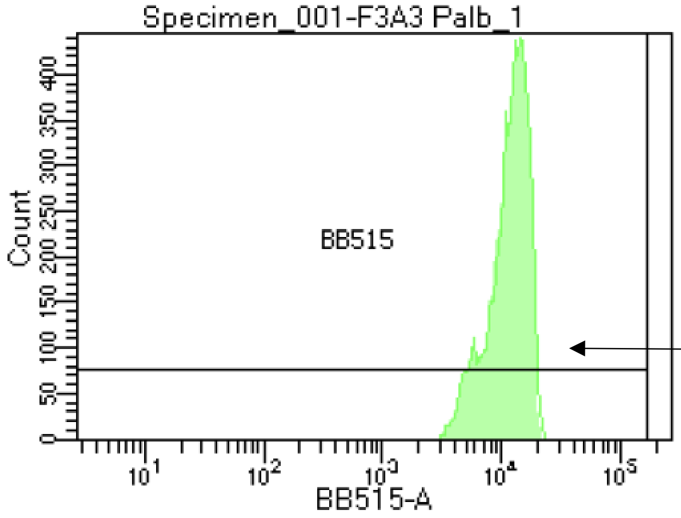

5, Arresting cells with 500 nM Palbociclib for 24 h lead to an increase of lipid droplet formation

Mean intensity: 11287

5. Cellular iron staining using Ferro-Orange

Example: Fig 5C

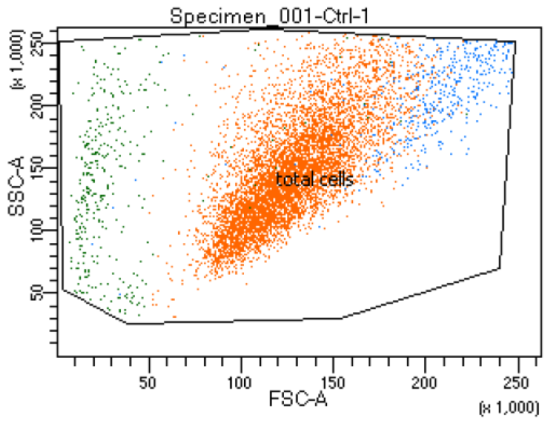

1, All cells are gated.

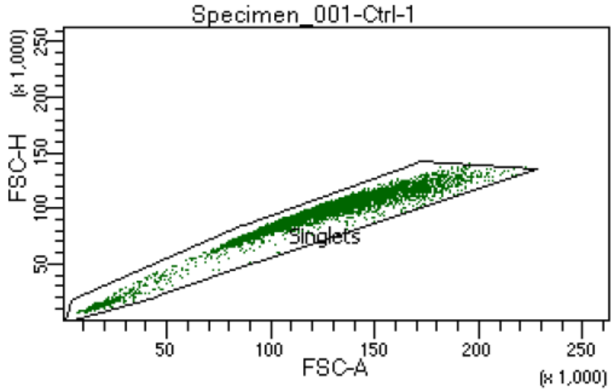

2, Single cells are gated.

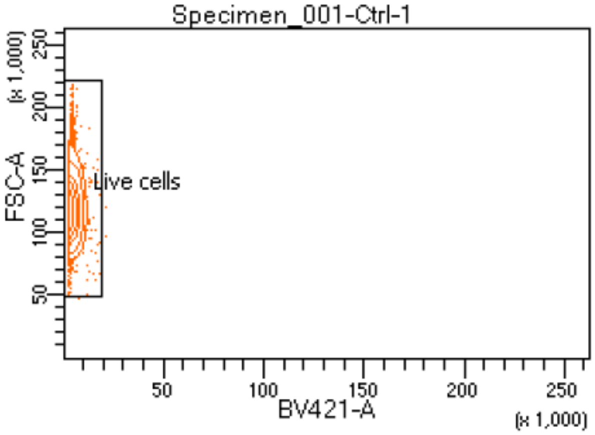

3, DAPI-negative cells were identified as live cells.

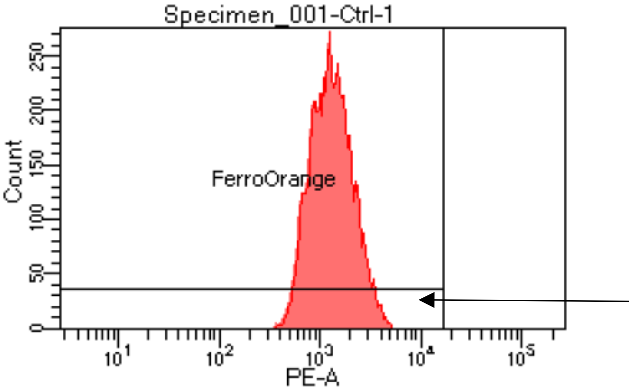

4, Cellular Fe^{2+} was stained with FerroOrange and analyzed with the PE detector

Mean intensity: 1375

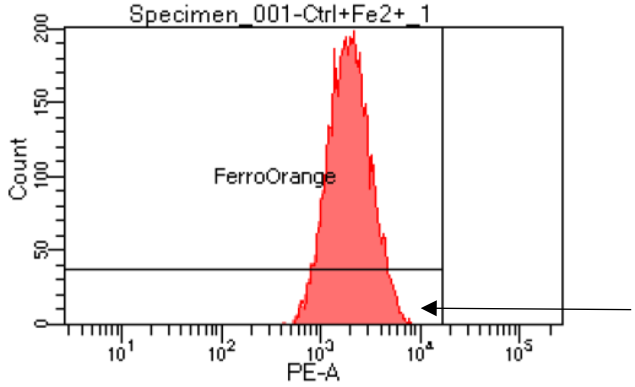

5, Addition of 100 uM Fe^{2+} in culture medium served as a positive control

Mean intensity: 2059
