# Supplementary material for: Activation of a FOXO3-induced cell cycle arrest regulates ferroptosis
Source: Cell Death Discov. 2025 Oct 16;11:465. doi: 10.1038/s41420-025-02760-x (PMC12533257; doi:10.1038/s41420-025-02760-x)

Supplementary material 2. Full-length and uncropped blots, related to Fig 1, 2, 3, 5 and S Fig 1B

Figure 1C

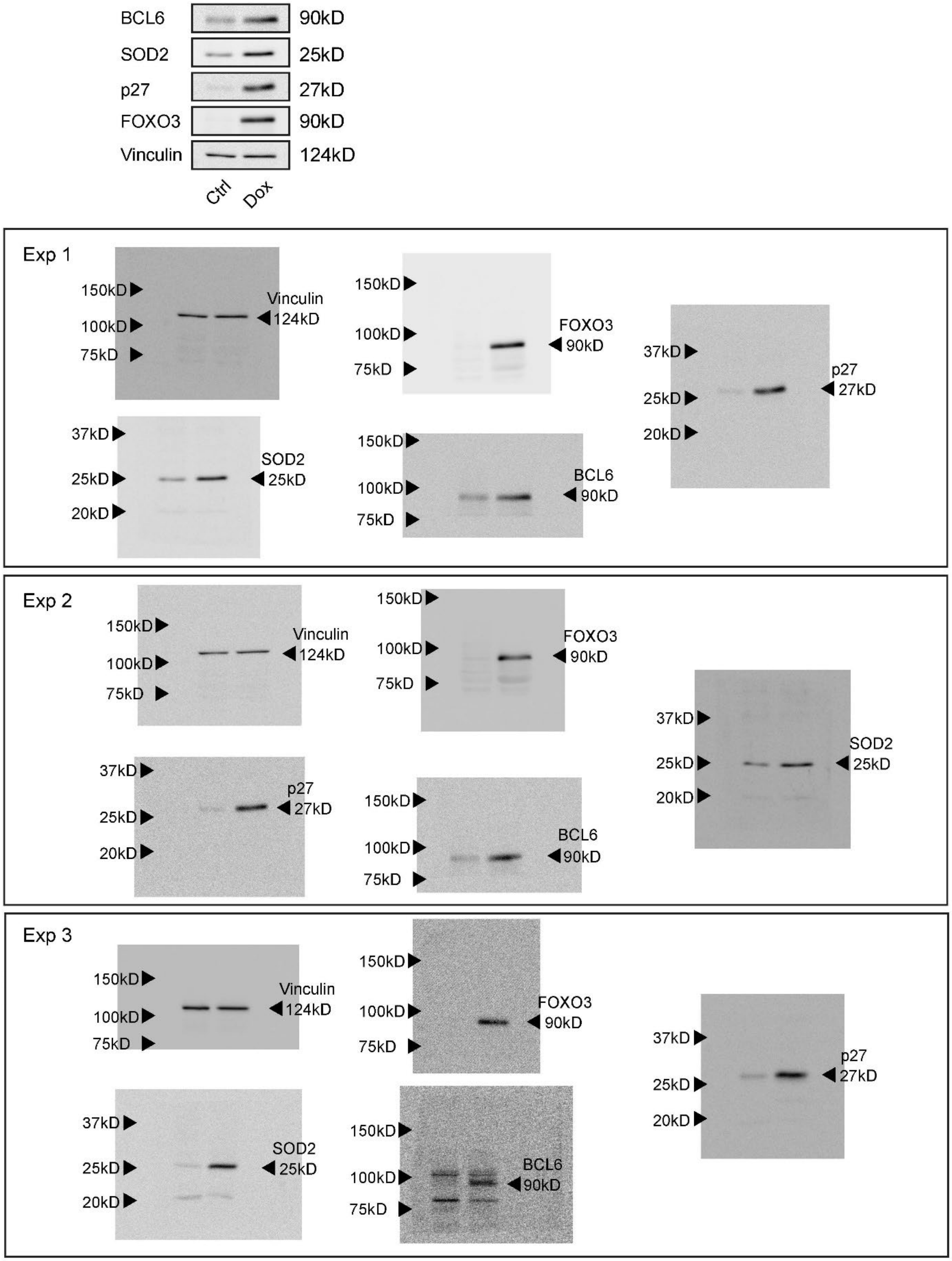

Figure 2A

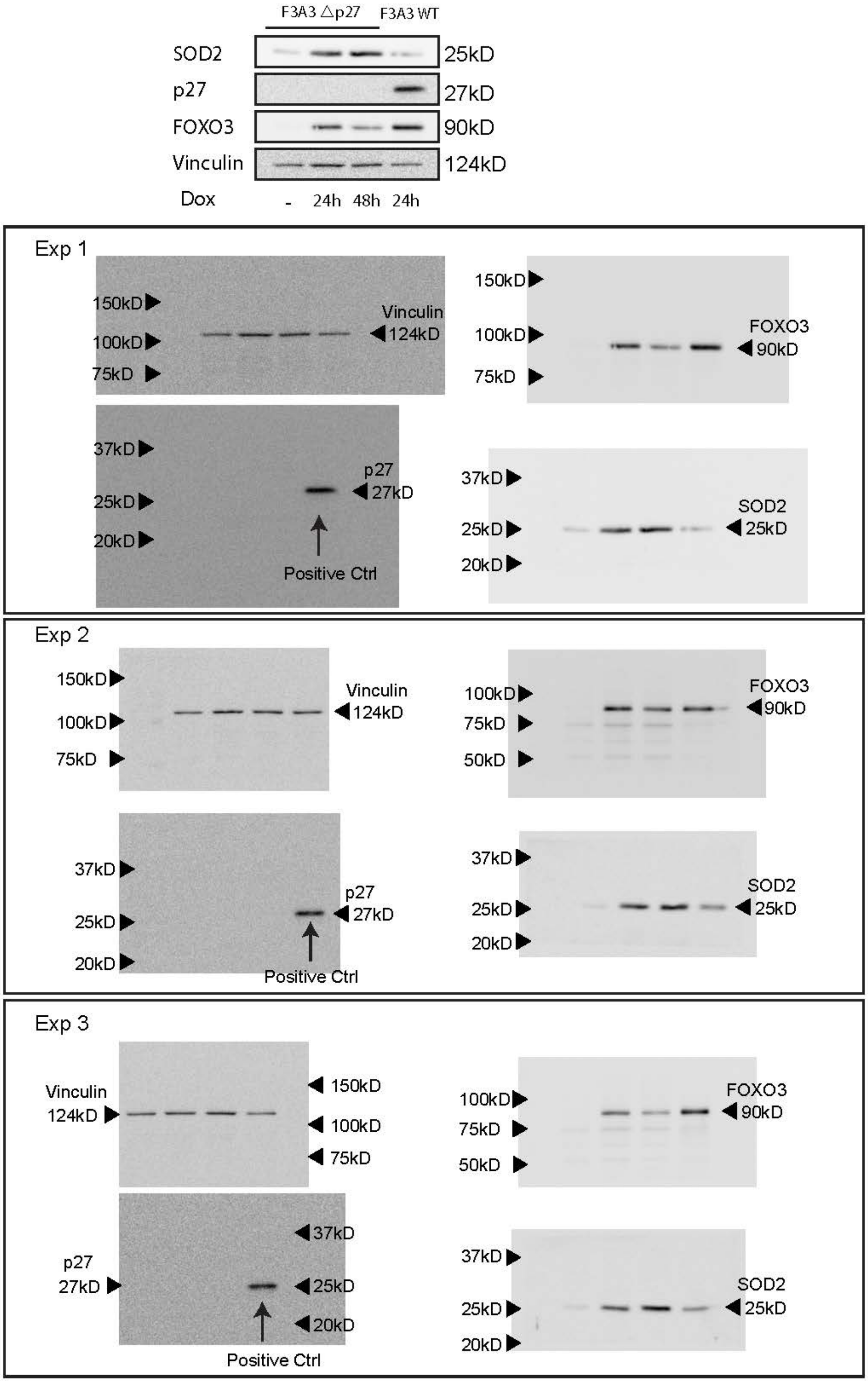
F3A3 △p27
F3A3 WT
SOD2
p27
FOXO3
Vinculin
25kD
27kD
90kD
124kD
Dox
-
24h
48h
24h
Exp 1
150kD
100kD
75kD
Vinculin
124kD
FOXO3
90kD
37kD
25kD
20kD
p27
27kD
Positive Ctrl
SOD2
25kD
Exp 2
150kD
100kD
75kD
Vinculin
124kD
100kD
75kD
50kD
FOXO3
90kD
37kD
25kD
20kD
p27
27kD
Positive Ctrl
SOD2
25kD
Exp 3
Vinculin
124kD
150kD
100kD
75kD
FOXO3
90kD
50kD
p27
27kD
37kD
25kD
20kD
Positive Ctrl
SOD2
25kD

Figure 3A

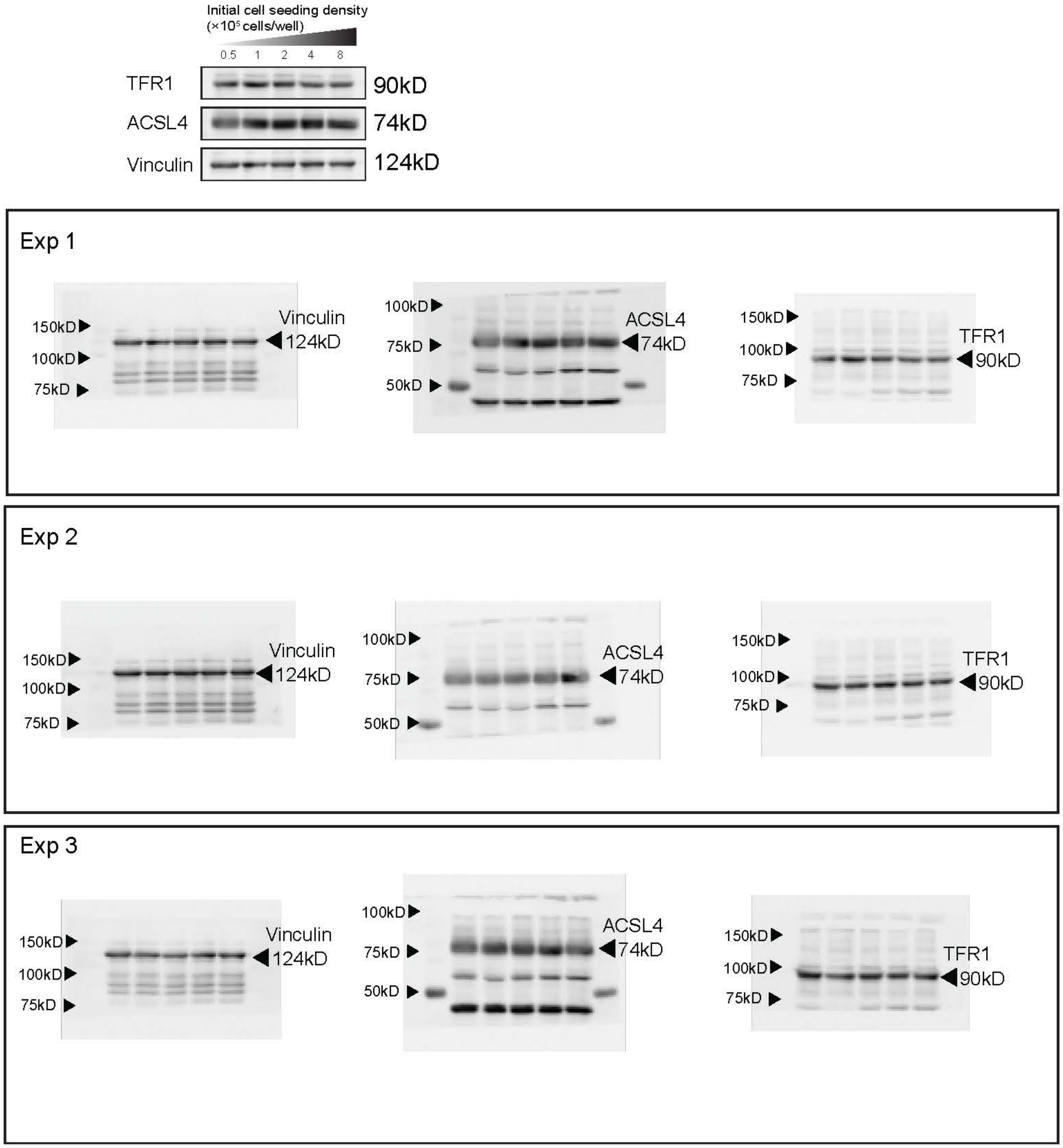

Initial cell seeding density
(×10⁵ cells/well)
0.5 1 2 4 8
TFR1
90kD
ACSL4
74kD
Vinculin
124kD
Exp 1
150kD
100kD
75kD
Vinculin
124kD
100kD
75kD
50kD
ACSL4
74kD
150kD
100kD
75kD
TFR1
90kD
Exp 2
150kD
100kD
75kD
Vinculin
124kD
100kD
75kD
50kD
ACSL4
74kD
150kD
100kD
75kD
TFR1
90kD
Exp 3
150kD
100kD
75kD
Vinculin
124kD
100kD
75kD
50kD
ACSL4
74kD
150kD
100kD
75kD
TFR1
90kD

Figure 3B

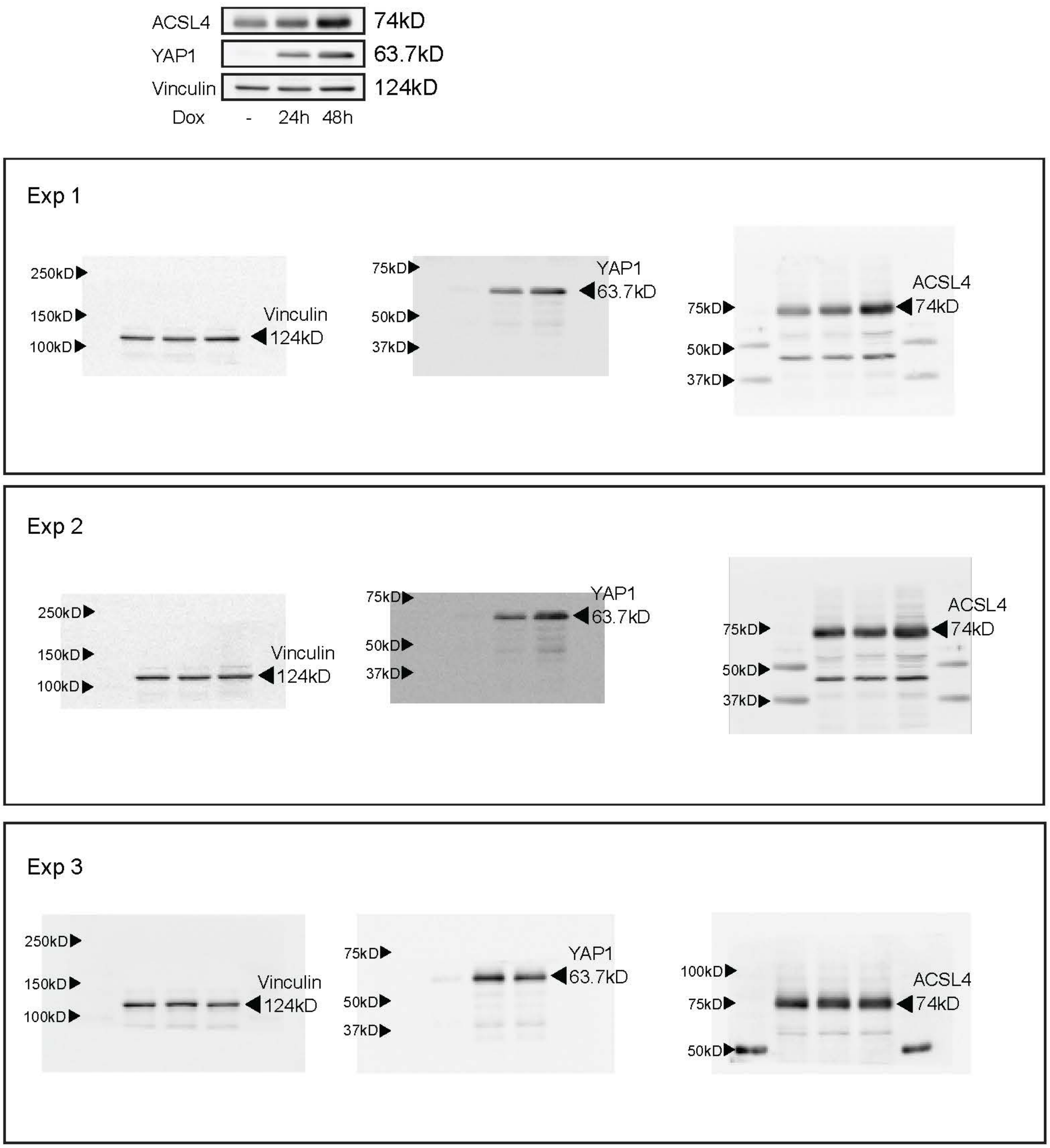

ACSL4
74kD
YAP1
63.7kD
Vinculin
124kD
Dox
-
24h
48h
Exp 1
250kD
150kD
100kD
Vinculin
124kD
75kD
50kD
37kD
YAP1
63.7kD
75kD
50kD
37kD
ACSL4
74kD
Exp 2
250kD
150kD
100kD
Vinculin
124kD
75kD
50kD
37kD
YAP1
63.7kD
75kD
50kD
37kD
ACSL4
74kD
Exp 3
250kD
150kD
100kD
Vinculin
124kD
75kD
50kD
37kD
YAP1
63.7kD
100kD
75kD
50kD
ACSL4
74kD

Figure 3C

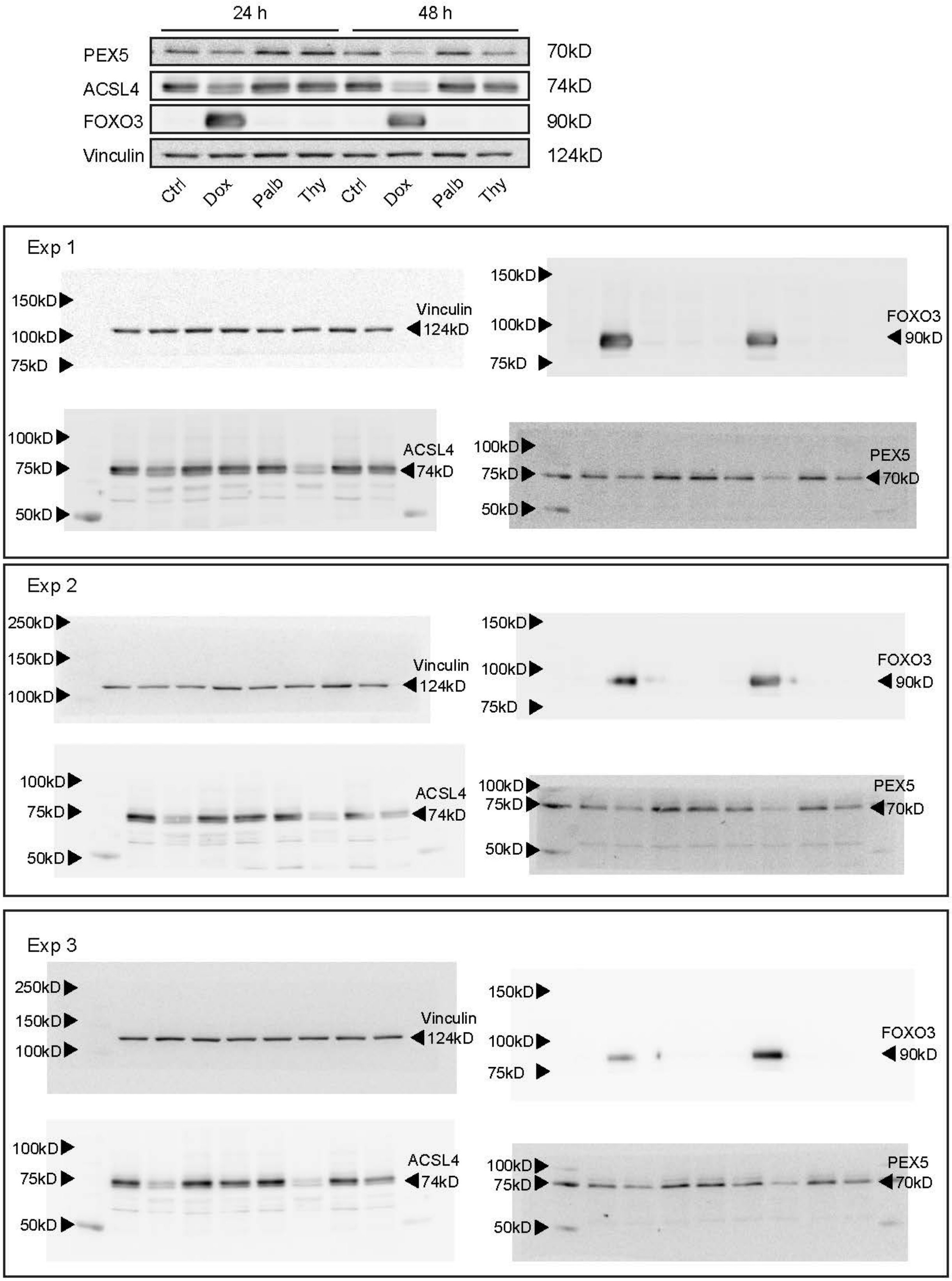

24 h
48 h
PEX5
ACSL4
FOXO3
Vinculin
70kD
74kD
90kD
124kD
Ctrl
Dox
Palb
Thy
Ctrl
Dox
Palb
Thy
Exp 1
150kD
100kD
75kD
Vinculin
124kD
150kD
100kD
75kD
FOXO3
90kD
100kD
75kD
50kD
ACSL4
74kD
100kD
75kD
50kD
PEX5
70kD
Exp 2
250kD
150kD
100kD
Vinculin
124kD
150kD
100kD
75kD
FOXO3
90kD
100kD
75kD
50kD
ACSL4
74kD
100kD
75kD
50kD
PEX5
70kD
Exp 3
250kD
150kD
100kD
Vinculin
124kD
150kD
100kD
75kD
FOXO3
90kD
100kD
75kD
50kD
ACSL4
74kD
100kD
75kD
50kD
PEX5
70kD

Figure 5A

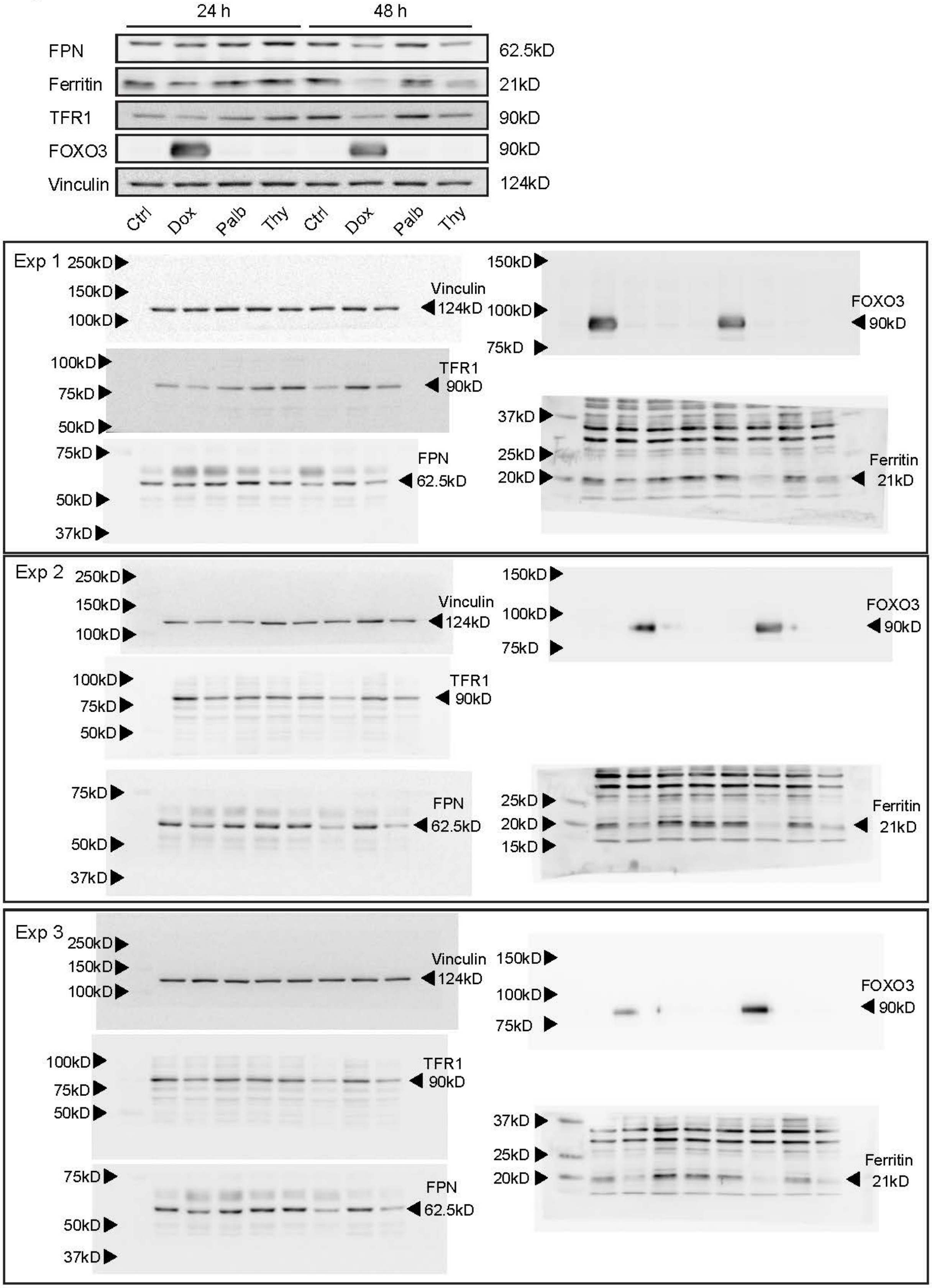
24 h
48 h
FPN
Ferritin
TFR1
FOXO3
Vinculin
62.5kD
21kD
90kD
90kD
124kD
Ctrl
Dox
Palb
Thy
Ctrl
Dox
Palb
Thy
Exp 1
250kD
150kD
100kD
Vinculin
124kD
100kD
75kD
50kD
TFR1
90kD
75kD
50kD
37kD
FPN
62.5kD
150kD
100kD
75kD
FOXO3
90kD
37kD
25kD
20kD
Ferritin
21kD
Exp 2
250kD
150kD
100kD
Vinculin
124kD
100kD
75kD
50kD
TFR1
90kD
75kD
50kD
37kD
FPN
62.5kD
150kD
100kD
75kD
FOXO3
90kD
25kD
20kD
15kD
Ferritin
21kD
Exp 3
250kD
150kD
100kD
Vinculin
124kD
100kD
75kD
50kD
TFR1
90kD
75kD
50kD
37kD
FPN
62.5kD
150kD
100kD
75kD
FOXO3
90kD
37kD
25kD
20kD
Ferritin
21kD

Figure 5F

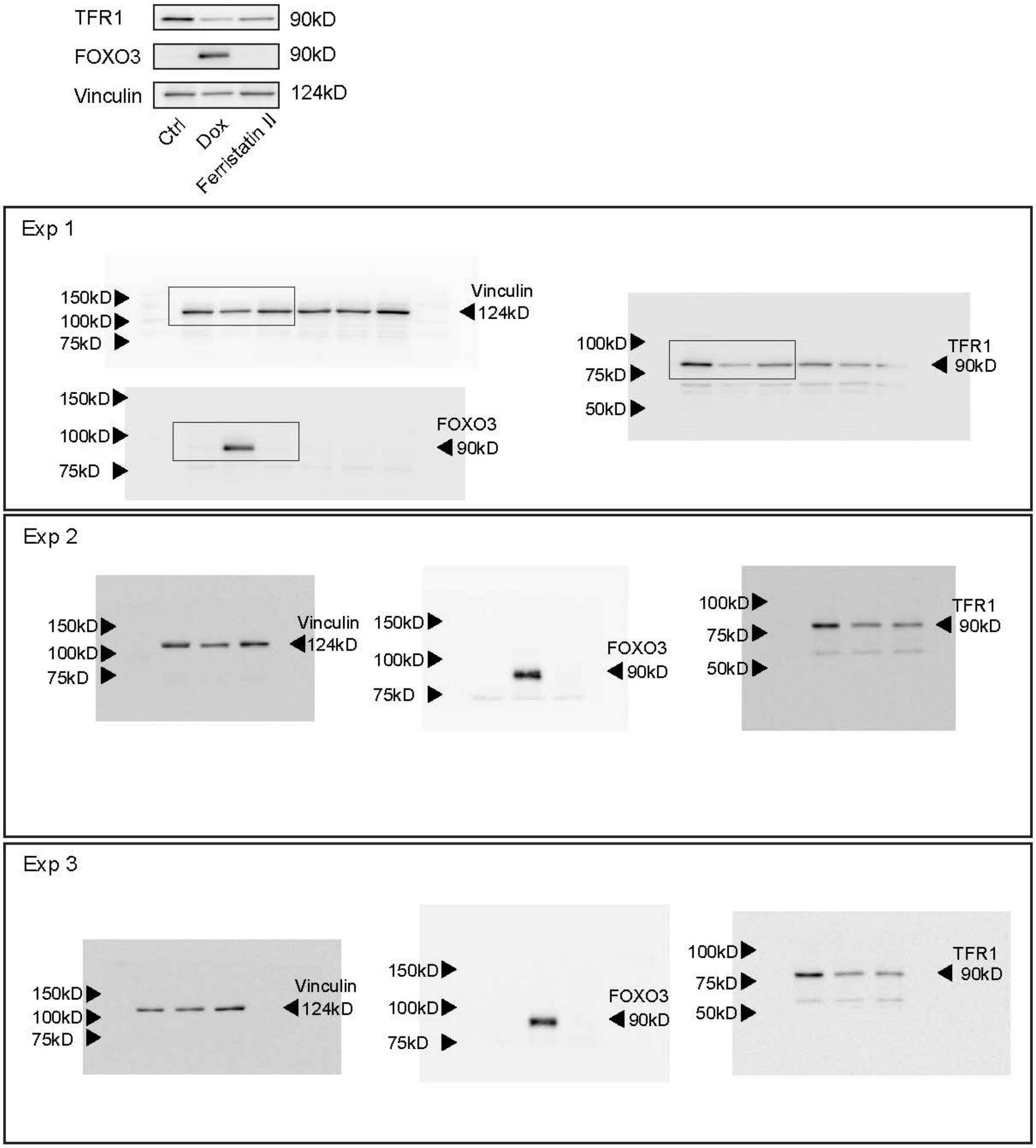

TFR1
FOXO3
Vinculin
90kD
90kD
124kD
Ctrl
Dox
Ferristatin II
Exp 1
150kD
100kD
75kD
Vinculin
124kD
150kD
100kD
75kD
FOXO3
90kD
100kD
75kD
50kD
TFR1
90kD
Exp 2
150kD
100kD
75kD
Vinculin
124kD
150kD
100kD
75kD
FOXO3
90kD
100kD
75kD
50kD
TFR1
90kD
Exp 3
150kD
100kD
75kD
Vinculin
124kD
150kD
100kD
75kD
FOXO3
90kD
100kD
75kD
50kD
TFR1
90kD

Data Supplement: Original images of representative western blot related to Supplementary Figure 1B

Experiment 1

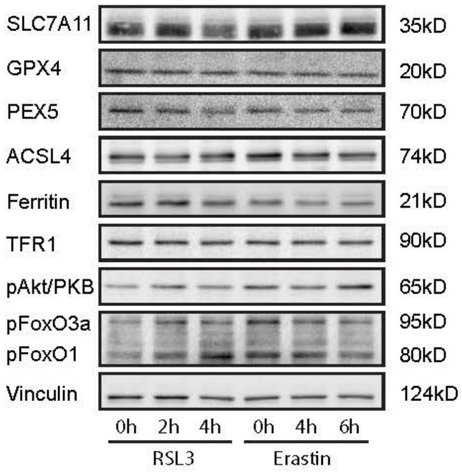

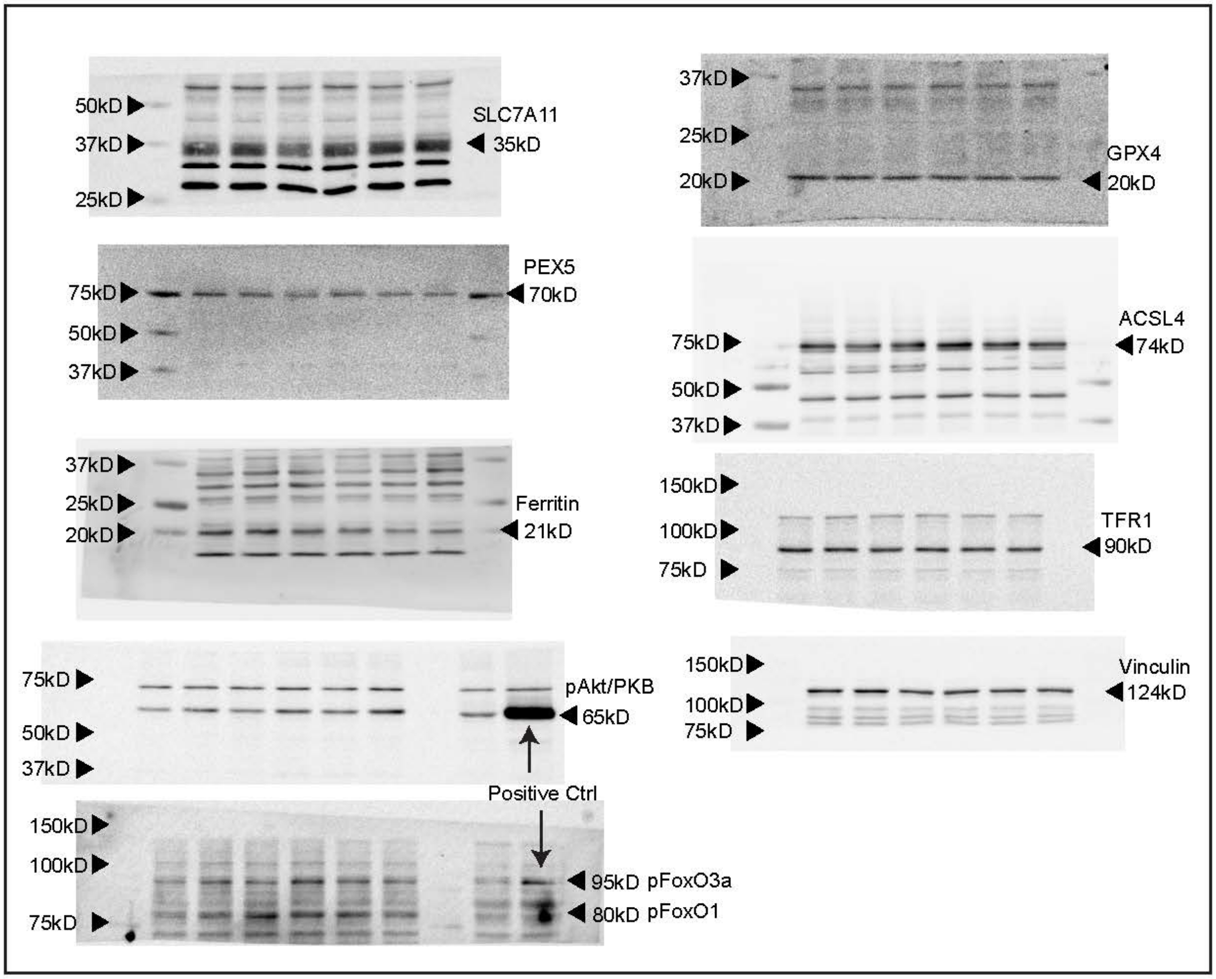

Supplementary Figure 1B

Experiment 2

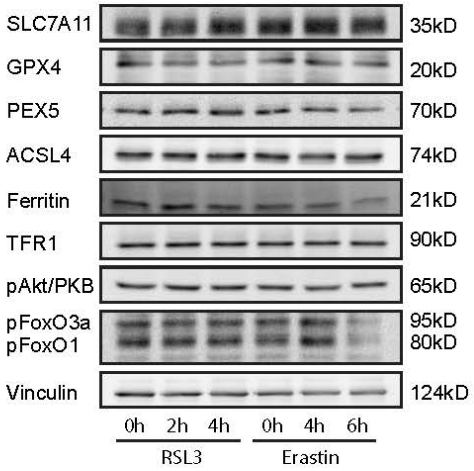

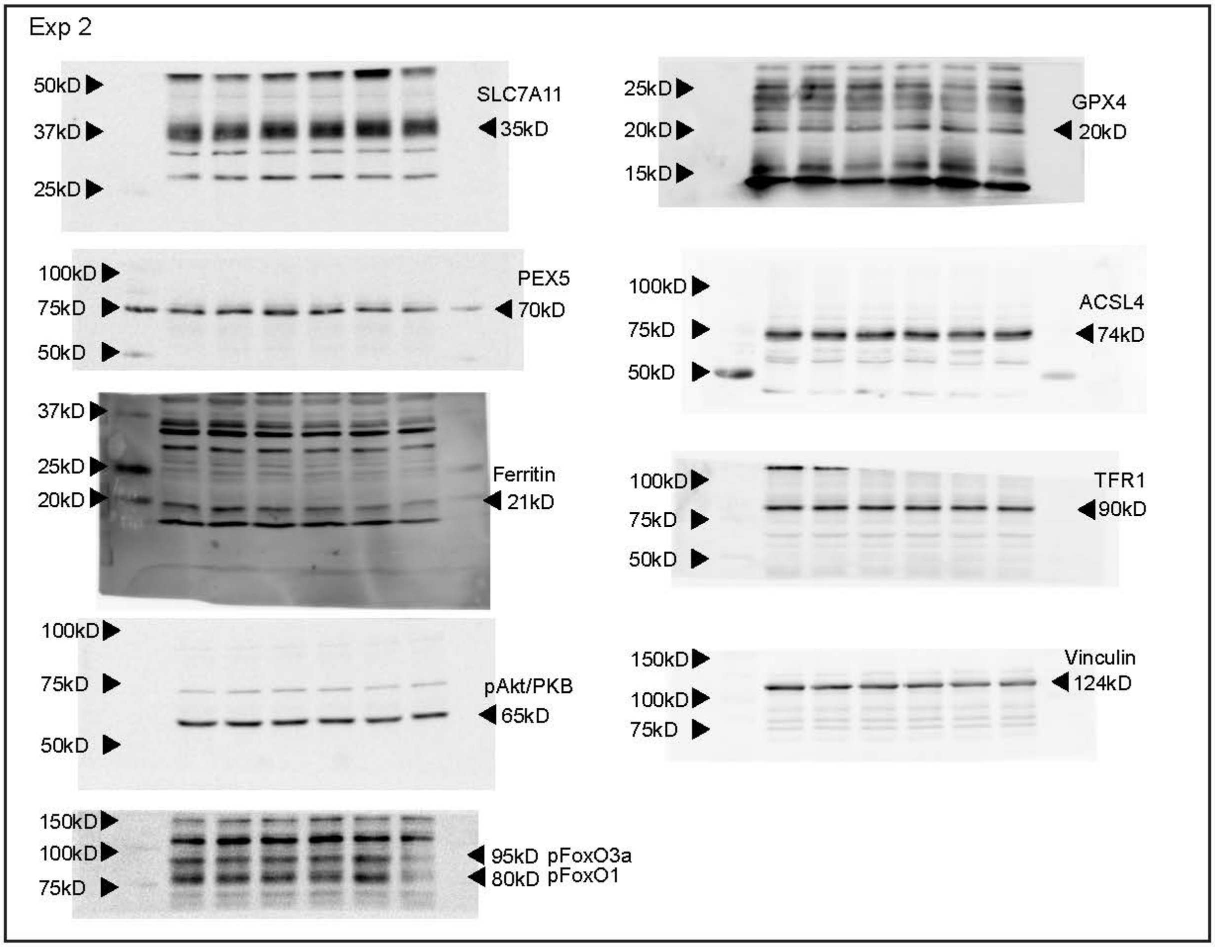

Supplementary Figure 1B

Experiment 3

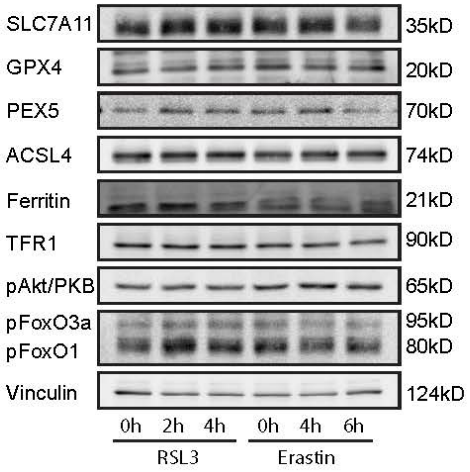

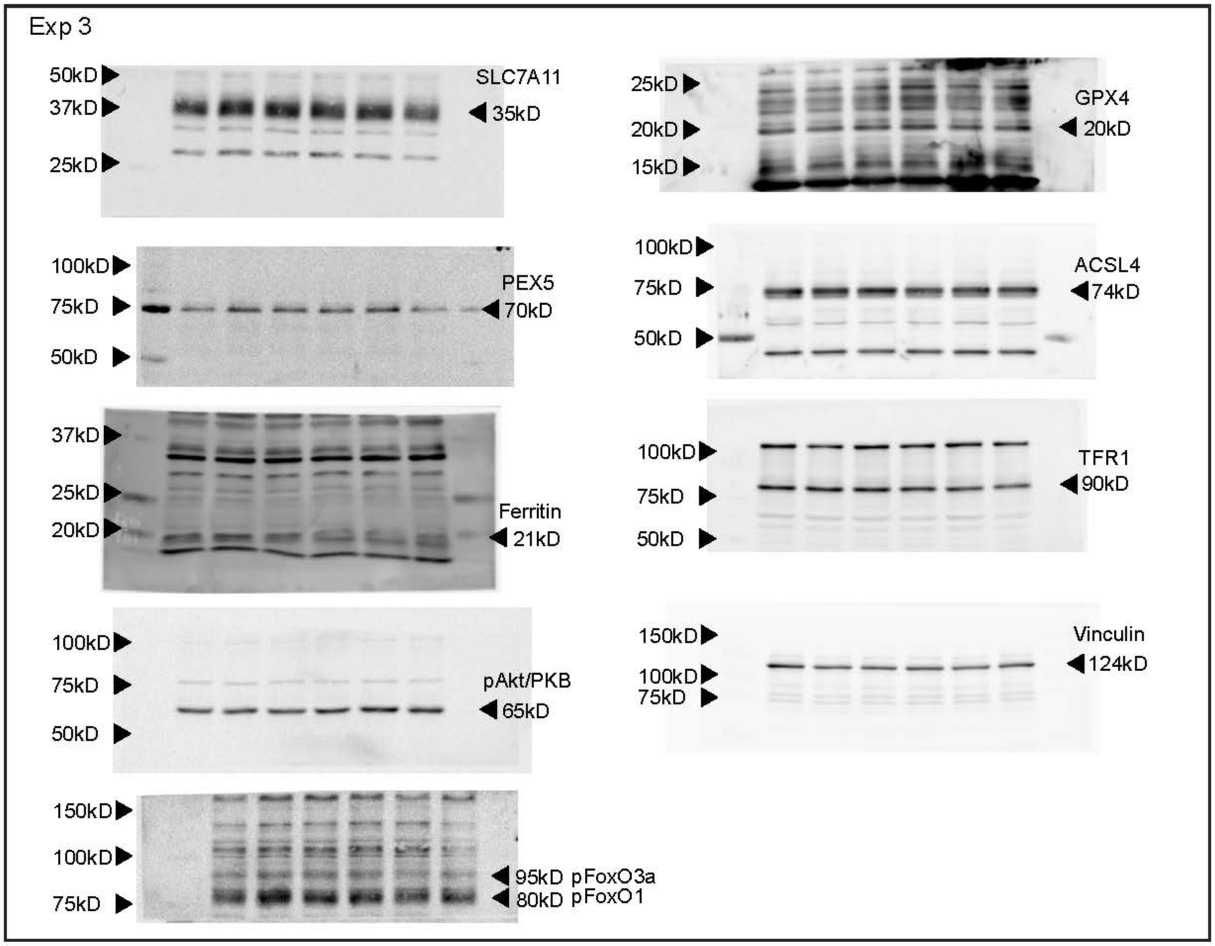

Supplement: Supplementary file 2 — Full-length and uncropped blots [file 41420_2025_2760_MOESM2_ESM.pdf]
